# Anti-Inflammatory Effects of Metformin Irrespective of Diabetes Status

**DOI:** 10.1161/CIRCRESAHA.116.308445

**Published:** 2016-08-18

**Authors:** Amy R. Cameron, Vicky L. Morrison, Daniel Levin, Mohapradeep Mohan, Calum Forteath, Craig Beall, Alison D. McNeilly, David J.K. Balfour, Terhi Savinko, Aaron K.F. Wong, Benoit Viollet, Kei Sakamoto, Susanna C. Fagerholm, Marc Foretz, Chim C. Lang, Graham Rena

**Affiliations:** From the Division of Molecular and Clinical Medicine, Ninewells Hospital and Medical School (A.R.C., D.L., M.M., C.F., C.B., A.D.M., A.K.F.W., C.C.L., G.R.) and Division of Neuroscience, Ninewells Hospital and Medical School (D.J.K.B.), MRC Protein Phosphorylation and Ubiquitylation Unit, College of Life Sciences (K.S.), University of Dundee, Scotland, United Kingdom; Institute of Biotechnology, University of Helsinki, Finland (V.L.M., T.S., S.C.F.); INSERM U1016, Institut Cochin, CNRS UMR8104, Université Paris Descartes, Sorbonne Paris Cité, France (B.V., M.F.); and Institute of Infection, Immunity, and Inflammation, University of Glasgow, United Kingdom (V.L.M.).

**Keywords:** cardiovascular diseases, diabetes mellitus, heart failure, inflammation, metabolism, metformin, NF-kappa B

## Abstract

**Rationale:**

The diabetes mellitus drug metformin is under investigation in cardiovascular disease, but the molecular mechanisms underlying possible benefits are poorly understood.

**Objective:**

Here, we have studied anti-inflammatory effects of the drug and their relationship to antihyperglycemic properties.

**Methods and Results:**

In primary hepatocytes from healthy animals, metformin and the IKKβ (inhibitor of kappa B kinase) inhibitor BI605906 both inhibited tumor necrosis factor-α–dependent IκB degradation and expression of proinflammatory mediators interleukin-6, interleukin-1β, and CXCL1/2 (C-X-C motif ligand 1/2). Metformin suppressed IKKα/β activation, an effect that could be separated from some metabolic actions, in that BI605906 did not mimic effects of metformin on lipogenic gene expression, glucose production, and AMP-activated protein kinase activation. Equally AMP-activated protein kinase was not required either for mitochondrial suppression of IκB degradation. Consistent with discrete anti-inflammatory actions, in macrophages, metformin specifically blunted secretion of proinflammatory cytokines, without inhibiting M1/M2 differentiation or activation. In a large treatment naive diabetes mellitus population cohort, we observed differences in the systemic inflammation marker, neutrophil to lymphocyte ratio, after incident treatment with either metformin or sulfonylurea monotherapy. Compared with sulfonylurea exposure, metformin reduced the mean log-transformed neutrophil to lymphocyte ratio after 8 to 16 months by 0.09 U (95% confidence interval, 0.02–0.17; *P*=0.013) and increased the likelihood that neutrophil to lymphocyte ratio would be lower than baseline after 8 to 16 months (odds ratio, 1.83; 95% confidence interval, 1.22–2.75; *P*=0.00364). Following up these findings in a double-blind placebo controlled trial in nondiabetic heart failure (trial registration: NCT00473876), metformin suppressed plasma cytokines including the aging-associated cytokine CCL11 (C-C motif chemokine ligand 11).

**Conclusion:**

We conclude that anti-inflammatory properties of metformin are exerted irrespective of diabetes mellitus status. This may accelerate investigation of drug utility in nondiabetic cardiovascular disease groups.

**Clinical Trial Registration:**

Name of the trial registry: TAYSIDE trial (Metformin in Insulin Resistant Left Ventricular [LV] Dysfunction). URL: https://www.clinicaltrials.gov. Unique identifier: NCT00473876.

## Abstract 2

Supplemental Digital Content is available in the text.

Metformin is the first-line drug in type 2 diabetes mellitus because compared with other type 2 diabetes mellitus treatments, in both clinical trials and in observational studies, metformin monotherapy is associated with fewer adverse cardiovascular events,^1,2^ and in some studies, a reduced risk of cancer.^3^ The reasons for this relative benefit are unclear, and metformin’s molecular action is a vigorous area of current research.^4–7^ Metformin’s chemical properties include a strongly hydrophilic character, metal-binding properties, and a pK_a_ within the physiological pH range.^6–8^ The key clinical hallmark of metformin’s antihyperglycemic action is suppression of hepatocyte gluconeogenesis.^4,5,9^ The most likely cellular effect underlying this response is inhibition of mitochondrial enzymes, including complex I in the electron transport chain.^10,11^ More recently, mitochondrial glycerophosphate dehydrogenase has been suggested as an alternative target.^12^ Mitochondrial inhibition activates AMP-activated protein kinase (AMPK),^13^ and recent work suggests that duodenal AMPK contributes toward effects of the drug on hepatic glucose production.^14^ Other studies indicate that metformin also suppresses glucose production by AMPK-independent mechanisms,^12,15,16^ but more broadly, AMPK may still contribute to metformin-dependent regulation of other aspects of metabolic control, such as lipogenic gene expression.^4^

The mechanism(s) underlying metformin’s advantage in incidence of cardiovascular disease (CVD) are unlikely to depend on effects of the drug on hyperglycemia, which is controlled equally well by metformin and insulin secretagogues.^2^ In addition, in animals, metformin suppresses infarct size and adverse remodeling in diabetic and nondiabetic rodents^17–21^ and retards heart failure progression in nondiabetic dogs.^22^ A better understanding of such glucose-independent properties might foster a more rational, less empirical exploitation of metformin in nondiabetic CVD. Inflammation, including nuclear factor-κB (NF-κB) signaling, is increasingly recognized as a significant contributing factor to diabetes mellitus (DM) and CVD,^23,24^ and several previous studies have found that metformin inhibits NF-κB signaling, including in vascular tissue^25^ and recently in hepatocytes.^26^ In the current study, we have used multiple approaches, including human studies, to define anti-inflammatory actions of metformin that may be separated from its antihyperglycemic action.

## Methods

### Animal and Cell Studies

Metformin and rapamycin came from calbiochem, 5-aminoimidazole-4-carboxamide riboside (AICAR) and A769662 (Tocris), tumor necrosis factor-α (TNF-α) (e-bioscience), recombinant CINC1/chemokine (C-X-C motif) ligand (CXCL) 1, C-C motif chemokine ligand (CCL)-11, interleukin (IL)-2, IL-4, stromal cell–derived factor and CCL22 (R&D systems), mouse IL-6 (Sigma), and recombinant mouse IL-1β (Life Technologies). The phospho–acetyl-CoA carboxylase Ser79 antibody was a generous gift from the DSTT (University of Dundee). The total acetyl-CoA carboxylase (Cat. number 3662), total AMPKα (2603), phospho-AMPKα Thr172 (2535), total S6 (2217), phospho-S6 Ser240/244 (2215), total p70 S6 kinase (2708), phospho-p70 S6 kinase Thr389 (9205), phospho-Raptor Ser 792 (2083), phospho inhibitor of kappa B kinase (IKK) α/β Ser176/177 (2078), IKKα/β Ser176/180 (2697), total IκB, pNF-κB, total IKKα, and total IKKβ (NF-κB sampler kit 9936) antibodies were from CST. Antisheep horseradish peroxidase (31480) and antirabbit horseradish peroxidase (31460) both came from Thermo and antimouse horseradish peroxidase was from Calbiochem (JA1200). BI605906 was generously gifted by Prof Sir Philip Cohen (Dundee).

### Animal Care

C57BL/6 female mice (Charles River; 8–41 weeks) were maintained under a 12 hours:12 hours light:dark cycle (holding room lights on at 06:00 and off at 18:00) at 22±1°C and 50% humidity. Mice had ad libitum access to standard chow diet (7.5% fat, 75% carbohydrate, and 17.5% protein by energy [RM1 diet; Special Diet Services]) and water. All animal care protocols and procedures were performed in accordance with current regulations.

### Cell Culture and Lysis for Immunoblotting

All cells were grown in an incubator at 37°C and 5% CO_2_. Primary mouse hepatocytes were extracted and maintained essentially as described previously.^6,15^

Bone marrow–derived macrophages (BMDMs) were grown from mouse bone marrow in RPMI 1640 medium supplemented with 10% fetal bovine serum (Life Technologies) and 10-ng/mL macrophage colony-stimulating factor (R&D systems). Cells were given fresh medium and growth factor on day 3 of culture. On day 6, BMDM cultures were supplemented with 100-ng/mL interferon γ (for M1 differentiation; R&D systems), 20-ng/mL IL-4 (for M2 differentiation; R&D systems), or 100-ng/mL lipopolysaccharide (for activation; premium grade from Sigma, expected to activate toll-like receptor [TLR]-2 and TLR4) in the presence or absence of drug treatments for the final 24 hours.

Before SDS-PAGE, cells were lysed by scraping into buffer A (50 mmol/L Tris acetate pH 7.5, 1% (wt/vol) Triton X100, 1 mmol/L EDTA, 1 mmol/L EGTA, 0.27 mol/L sucrose, 50 mmol/L NaF, 1 mmol/L sodium orthovanadate, 10 mmol/L β-glycerophosphate, 5 mmol/L sodium pyrophosphate, 1 mmol/L benzamidine, 0.2 mmol/L phenylmethylsulfonyl fluoride, and 0.1% (v/v) β-mercaptoethanol) and then prepared for SDS-PAGE as described in the previous work.^6^ Immunoblot densitometry for each antibody was performed with Image Studio Lite version 5.2 (LI-COR). Each blot is representative of experiments preformed at least 3×.

### Glucose Assay

Treatment of cells for hepatocyte glucose production was performed essentially as described previously, using primary mouse hepatocytes plated in 12-well plates (1.25×10^5^ cells per well).^6,15,27^ Glucose production was determined after a 12-hour incubation period in glucose-free DMEM (11966; Life Technologies) supplemented with 1% pen/strep, lactate (Sigma)/pyruvate (Life Technologies; 10:1 mmol/L), and 100 nmol/L dexamethasone (dex; Merck) with or without drugs/cytokines under investigation. At the end of the incubation period of 12 hours, 500 μl of medium was collected and glucose concentration determined by GAGO assay (glucose [glucose oxidase]; Sigma) by a modified protocol scaled down to a 96-well plate format. Each column consists of data from at least 12 wells of cells, 6 each from 2 mice.

### Real-Time-Polymerase Chain Reaction

Total RNA from primary mouse hepatocytes was extracted using QIAshredder (Qiagen) and Rneasy MINI KIT (Qiagen). cDNA was synthesized using RQ1 Rnase-Free Dnase kit (Promega) and ImProm-II Reverse Transcription System (Promega). Nucleospin RNA II Total RNA isolation kit (Macherey-Nagel) was used to isolate RNA from macrophages. cDNA was synthesized using High Capacity cDNA Reverse Transcription Kit (4368814, Thermo Fisher Scientific). Real-time polymerase chain reaction was performed using the 7900HT Fast Real-Time PCR System (Applied Biosystems) using TaqMan 2× Universal PCR Master Mix (Applied Biosystems) and primer/probes mixes as stated (Applied Biosystems). Primer sets used were as follows: IL-6 Mm00446190_m1, CXCL1 Mm04207460_m1, 18S Hs03003631_g1, IL-1β Mm00434228_m1, CXCL2 Mm00436450_m1, peroxisome proliferator–activated receptor-γ m01184322_m1, fatty acid synthase Mm00662319_m1, CCL22 Mm00436439_ml, CXCL12 Mm00445553_ml, TATA-binding protein Mm01277042_m1, and sterol regulatory element-binding protein 1c Mm00550338_m1. Cycling conditions were as follows: 50°C for 2 minutes, 95°C for 10 minutes, followed by 40 cycles of 95°C for 15 s and 60°C for 1 minute. Expression is expressed relative to 18s mRNA for hepatocytes and TATA-binding protein for macrophages (Applied Biosystems) using the 2^-ΔΔCt^ method. Each column is composed of data from at least 3 separate experiments.

### BMDM Analysis

BMDMs were harvested from culture plates using 4 mmol/L EDTA in PBS for 10 minutes at 37°C. Cells were washed in flow cytometry buffer (PBS with 2% fetal bovine serum and 1 mmol/L EDTA) and stained using the following antibodies (all BD Bioscience unless stated): F4/80 (BM8; e-bioscience), CD11c (HL3), CD206 (C068C2; Biolegend), CD69 (H1.2F3), and CD40 (3/23). Fc block (4.4G2) was included in all stains. Data were acquired on a LSR II flow cytometer (Becton Dickinson) and analyzed using FlowJo software (TreeStar). BMDM culture supernatants were collected after 24-hour treatment with the differentiation or activation conditions. Levels of cytokines were quantified by standard sandwich ELISA using paired antibody kits (e-bioscience).

### Validation in Clinical Patients

We validated the animal study findings in clinical patients utilizing 2 approaches: a retrospective population cohort study and a randomized placebo-controlled study of metformin. All patients provided written informed consent to participate in these clinical studies that were approved the local ethics committee.

### Population Cohort Study: Metformin Exposure in DM Patients and Neutrophil to Lymphocyte Ratio.

In the population cohort study, we investigated whether the anti-inflammatory signature of metformin could be detected in humans with DM, using the GoDARTS (Genetics of Diabetes Audit and Research in Tayside Scotland) DM register.^28^ We compared the effect of metformin and sulfonylureas on the neutrophil to lymphocyte ratio (NLR), a marker of inflammation derived from a combination of hematological components of the systemic inflammatory response^29,30^ that has recently been found to be a predictor of all-cause mortality and cardiac events.^31^ We analyzed data from type 2 diabetes mellitus patients recruited in Tayside, Scotland, UK, between October 1, 1997, and March 1, 2010. Of the 9205 subjects with DM within the GoDARTS study, we chose 3575 treatment naive patients who were either incident metformin users or incident sulfonylurea users (but not both) and noninsulin users. Incident use meant at least 6 months before first observed metformin/sulfonylurea prescription date during which they were observable for drugs. Of these 670 patients (mean [SD]: age, 65 [11] years; 54% men) had derived NLR values both at baseline (up to 120 days before first metformin/sulfonylurea prescription) and follow-up (8–16 months after baseline). NLR was calculated as the ratio of the neutrophil:lymphocyte count, both obtained from the same blood sample. A total of 498 (74%) patients were treated with metformin and 172 (26%) with sulfonylurea. Multivariate linear and logistic regression models were run on the 8- to 16-month follow-up NLR against the treatment group, controlling for covariates including age, sex, and baseline NLR value.

### Randomized Placebo-Controlled Study: Metformin Exposure and Cytokine Levels in Nondiabetic Heart Failure Patients

The anti-inflammatory effects of metformin were investigated in a randomly selected subset of patients who had participated in a double-blind, placebo-controlled study (www.clinicaltrials.gov: NCT00473876) that had evaluated the impact of metformin on insulin resistant (IR) and exercise capacity in nondiabetic patients with congestive heart failure.^32^ Every patient had provided written informed consent before participation in this study, which was approved by the East of Scotland Research Ethics Service. The subset of patients selected for this study involved 33 nondiabetic IR congestive heart failure patients (mean age, 63±7.0 years; men, 85%; New York Heart Association class I/II/III/IV, 04/28/01/0) who were randomized to receive either 4 months of metformin (n=20; 2 g/d) or matching placebo (n=13). IR was defined by a fasting insulin resistance index (FIRI) of ≥2.7. The effect of metformin on plasma inflammatory cytokines was examined by investigating changes from baseline to final visit after 4 months in the study.

### Cytokine Assay

Human plasma was analyzed using the Bio-Plex Pro Human Chemokine 40-Plex Panel (171-AK99MR2, Bio-Rad). The assay was performed following the manufacturer’s instructions using the Bio-Plex 200 system (Bio-Rad). Freeze–thaw cycling of samples was avoided to prevent cytokine degradation, and they were diluted 1:4 (12.5 μL of plasma) for the assay.

### Statistical Analyses

Results in bar graphs are expressed as mean±SEM. Comparisons between groups were made by 1-way ANOVA with Dunnett or Tukey post hoc test using Prism. Differences were considered statistically significant if *P*<0.05: ***P<0.001, **P<0.01, and *P<0.05 unless otherwise stated. For studies on the plasma, statistical analyses of data were performed using SPSS 14.1. ANOVA and Pearson correlation coefficients were calculated.

## Results

### Metformin Inhibits TNF-α–Dependent NF-κB Inflammatory Signaling, Comparably With the Specific IKKβ Inhibitor BI605906

**Figure 1.**
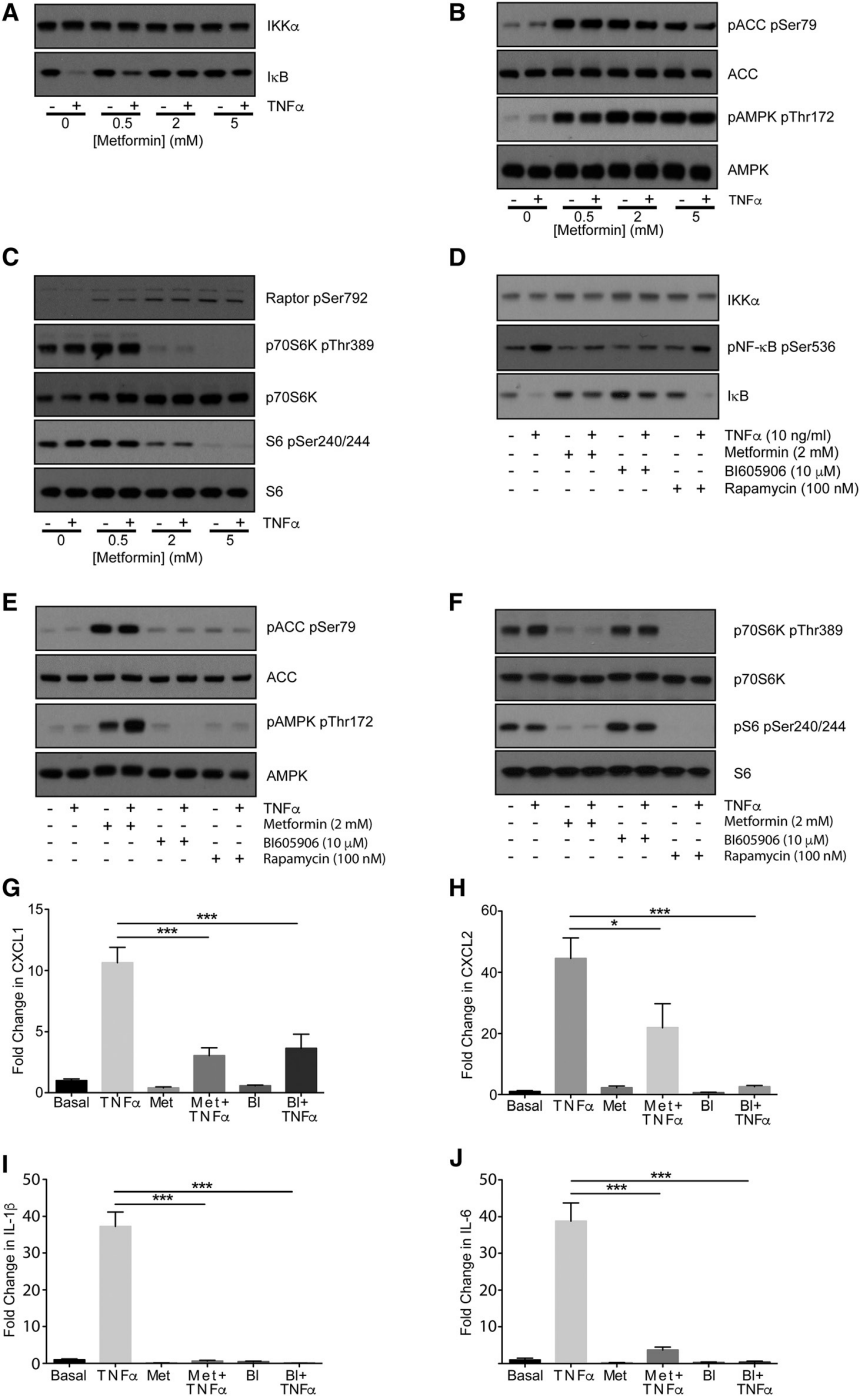
**Effect of metformin on nuclear factor**-κ**B (NF-κB) signaling and gene expression**. **A**–**C**, Primary hepatocytes were incubated in serum-free medium overnight and then stimulated for 3 h with or without 0.5 to 5 mmol/L metformin. For the last 15 min, cells were treated with or without 10 ng/mL tumor necrosis factor (TNF)-α. Cells were lysed and prepared for immunoblotting using antibodies as described in the Methods section of this article. In this figure and elsewhere, each blot is representative of experiments carried out at least 3×. **D**–**F**, Primary hepatocytes were incubated as in **A**–**C**, before stimulation for 3 h with or without 2 mmol/L metformin and TNF-α. In addition, cells were incubated with/without 10 μmol/L BI605906 or 100 nmol/L rapamycin as shown, before lysis and immunoblotting as described in the Methods section of this article. **G**–**J**, Primary hepatocytes were treated with or without 10 ng/mL TNF-α, 2 mmol/L metformin, or 10 μmol/L BI605906 for 8 h followed by cell lysis, RNA extraction, and preparation of cDNA for real-time-polymerase chain reaction using primer sets for individual genes shown as described in the Methods section of this article. ACC indicates acetyl-CoA carboxylase; AMPK, AMP-activated protein kinase; p-ACC, phospho–acetyl-CoA carboxylase; and pAMPK, phospho–AMP-activated protein kinase.

In primary mouse hepatocytes, the main target of metformin’s antihyperglycemic effects, we compared metformin with the specific IKKβ inhibitor BI605906.^33^ Metformin treatment for 3 hours suppressed TNFα-induced degradation of the NF-κB negative regulator IκB, while modulating AMPK and mammalian target of rapamycin signaling in a dose-dependent manner (Figure 1A through 1C; all densitometry appears in the Online Data Supplement). The magnitude of the effect on IκB was comparable with BI605906 (Figure 1A and 1D). Unlike metformin, BI605906 did not suppress signaling downstream of mammalian target of rapamycin nor did it activate AMPK (Figure 1E and 1F). We were unable to detect any effect of rapamycin on NF-κB signaling either (Figure 1D), suggesting that the effect of metformin on NF-κB and mammalian target of rapamycin occurs independently. Consistent with these signaling results, TNF-α–dependent expression of CINC-1/CXCL1, CXCL2, IL-1β, and IL-6 was strongly inhibited by both metformin and BI605906 (Figure 1G through 1J).

### AMPK-Independent Regulation of NF-κB in Primary Hepatocytes

**Figure 2.**
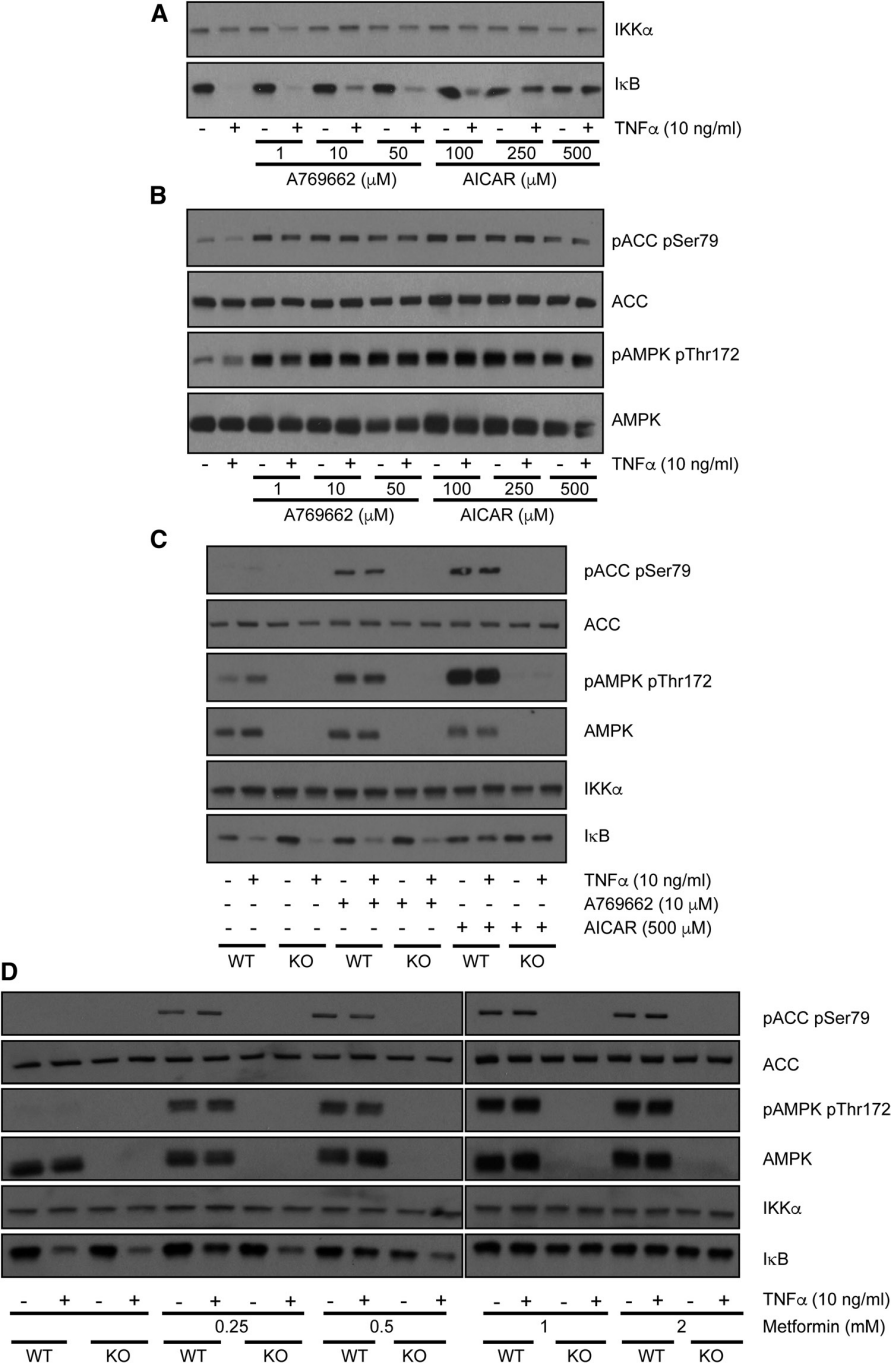
**Effect of 5-aminoimidazole-4-carboxamide riboside (AICAR) and A769662 on nuclear factor**-κ**B (NF-κB) signaling**. **A**–**D**, Primary wild-type (WT) hepatocytes (**A** and **B**) and those taken from double-knockout (KO) AMPK animals or matched controls (WT) (**C**) were incubated in serum-free medium overnight, before stimulation for 3 h with or without doses of A769662 and AICAR as shown. For the last 15 min, cells were treated with or without 10 ng/mL tumor necrosis factor (TNF)-α. **D**, Hepatocytes from KO or WT animals treated with and without doses of metformin for 3 h. For the last 15 min, cells were treated with 10 ng/mL TNF-α. Cells were then lysed, and immunoblots were prepared as described in the Methods section of this article and in Figure 1. ACC indicates acetyl-CoA carboxylase; AMPK, AMP-activated protein kinase; IKK, inhibitor of kappa B kinase; p-ACC, phospho–acetyl-CoA carboxylase; and pAMPK, phospho–AMP-activated protein kinase.

To determine whether metformin directly regulated kinase activity that may mediate its effects on NF-κB signaling, a cell-free kinase profiling assay was performed. Metformin did not directly inhibit the upstream NF-κB regulator IKKβ, and most other kinases exhibited little, if any, inhibition by metformin and none were inhibited >50% (data available on the profiling website http://www.kinase-screen.mrc.ac.uk/). These results suggest that metformin is unlikely to exert effects on NF-κB through direct IKKβ inhibition or inhibition of other kinases. The lack of effect of metformin on kinase activity led us to explore the possibility that IκB regulation might occur as a consequence of AMPK activation,^13^ which occurs after mitochondrial inhibition by the drug.^10,11^ In side-by-side experiments, we treated primary hepatocytes with AICAR (an AMP mimetic) and A769662, a direct AMPK activator. Compared with AICAR, which suppressed IκB degradation, there was little, if any, effect of A769662 on IκB degradation at the doses used (Figure 2A), but both agents induced phosphorylation of the AMPK substrate acetyl-CoA carboxylase (Figure 2B). To investigate possible reason(s) for this difference, we investigated primary liver cells from AMPK catalytic subunit-deficient mice.^15^ In these cells, AICAR still suppressed IκB degradation (Figure 2C), suggesting that AICAR effect is AMPK independent. Consistent with this, the effect of metformin on IκB signaling was similar in both genotypes (Figure 2D).

### Dissociation of Anti-Inflammatory Responses From Effects of Metformin on Hepatic Glucose Production and Lipogenic Gene Expression

**Figure 3.**
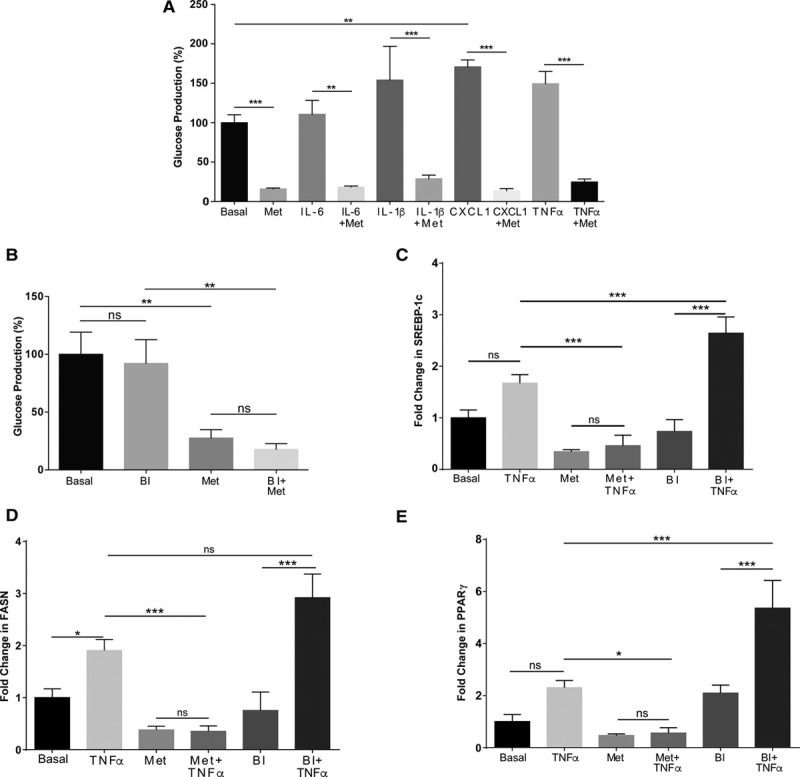
**Effects of cytokines on glucose production and lipogenic gene expression in primary hepatocytes. A** and **B**, Primary hepatocytes were treated with/without metformin (2 mmol/L), interleukin (IL)-6 (5 ng/mL), IL-1β (10 ng/mL), chemokine (C-X-C motif) ligand (CXCL) 1 (100 ng/mL) and tumor necrosis factor (TNF)-α (10 ng/mL) for 12 h, and glucose production was measured by GAGO (glucose [glucose oxidase]) assay as described in the Methods section of this article. **C**–**E**, Primary hepatocytes were treated with or without 10 ng/mL tumor necrosis factor (TNF)-α, 2 mmol/L metformin, and 10 μmol/L BI605906 for 8 h followed by cell lysis, RNA extraction, and preparation of cDNA for real-time polymerase chain reaction using primer sets for individual genes shown as described in the Methods section of this article. FASN indicates fatty acid synthase; PPAR, peroxisome proliferator–activated receptor; and SREBP, sterol regulatory element-binding protein.

Metformin’s main antihyperglycemic effect is to reduce hepatic glucose production. To determine whether metformin-regulated cytokines directly altered glucose production, we incubated hepatocytes with and without metformin, IL-6, IL-1β, CXCL1, and TNFα. CXCL1 significantly increased glucose production (Figure 3A). In all groups, metformin reduced glucose production to below control levels (basal) in the presence or absence of cytokine. Incubation of hepatocytes with BI605906 did not mimic the effect of metformin, nor was there any modulation of metformin’s suppression of glucose production (Figure 3B).

Next, we compared the effect of metformin and BI605906 on lipogenesis, which is an another metabolic response known to be regulated by metformin. Proinflammatory cytokines including TNF-α are known to induce lipogenesis.^34^ This prompted us to study the effects of TNF-α on lipogenic genes sterol regulatory element-binding protein 1c, peroxisome proliferator–activated receptor-γ, and fatty acid synthase, which are known to be regulated by metformin in hepatocytes.^13,32,35^ TNF-α significantly increased fatty acid synthase mRNA expression, with a trend toward increased expression of sterol regulatory element-binding protein 1c and peroxisome proliferator–activated receptor-γ (Figure 3C through 3E). Metformin reduced mRNA expression of all 3 genes and prevented TNF-α–induced increases. In contrast to the inflammatory genes, coincubation of BI605906 and TNF-α increased lipogenic gene expression (Figure 3C through 3E). BI605906 alone did not alter sterol regulatory element-binding protein 1c, fatty acid synthase, or peroxisome proliferator–activated receptor-γ; however, this compound significantly augmented TNF-α–induced expression of each gene. This may be related to the existence of negative feedback loops in NF-κB signaling.^33^

### Direct Anti-Inflammatory Effect of Metformin on Macrophage Cytokine Secretion

**Figure 4.**
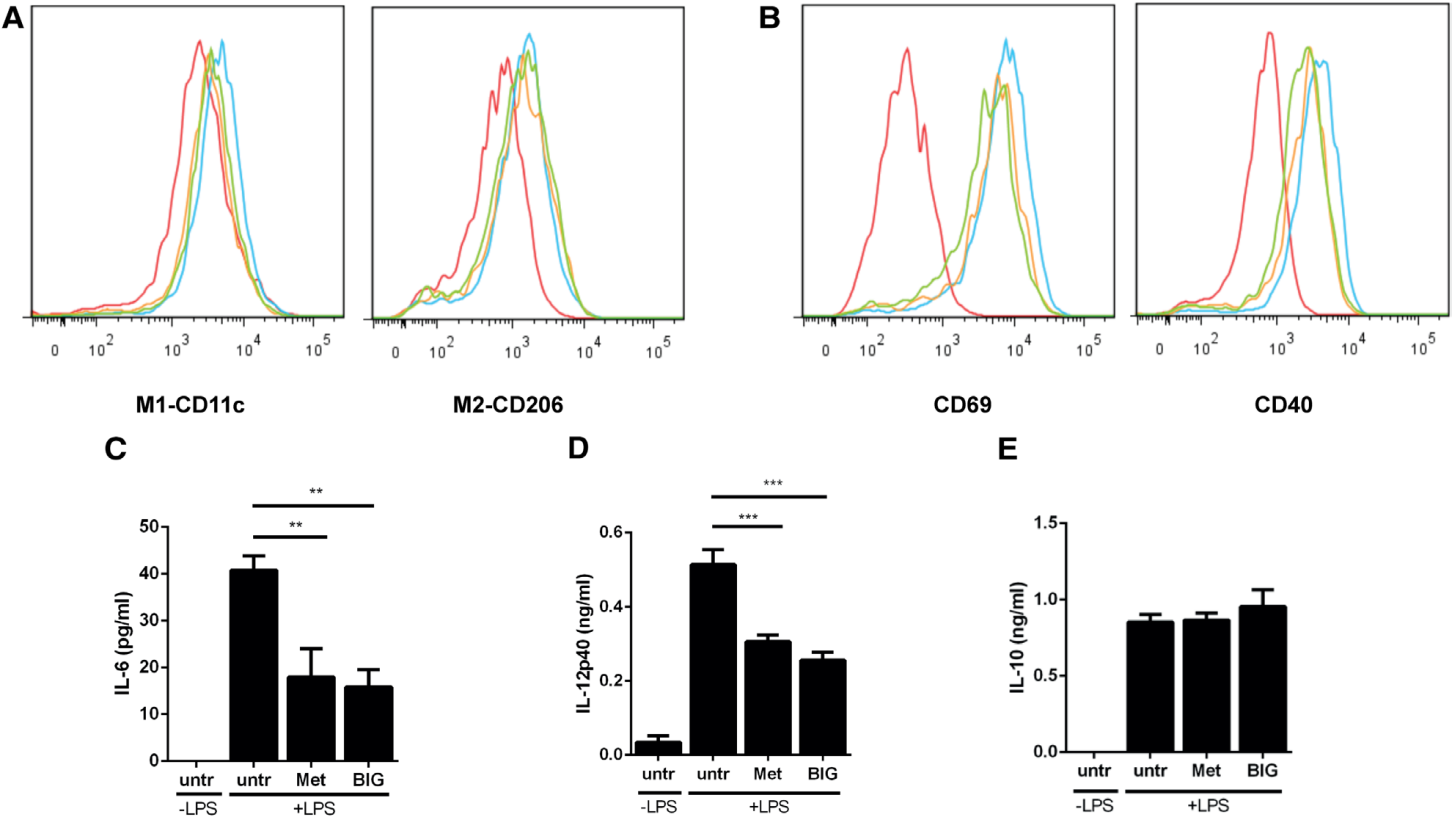
**Effect of metformin and its analogue biguanide on bone marrow–derived macrophages: phenotypic markers and cytokine secretion**. **A**, Macrophages were treated with/without metformin (2 mmol/L) or biguanide (BIG 2 mmol/L) to determine the effect on the M1 and M2 phenotypes of macrophages, which was measured by flow cytometry for CD11c and CD206 expression. The colors denote the following: red, undifferentiated; blue, differentiated, untreated; orange, differentiated, metformin; green, differentiated, BIG. **B**, Macrophages were treated with/without metformin (2 mmol/L) or biguanide (BIG, 2 mmol/L) to determine the effect on activation in response to 100 ng/mL lipopolysaccharide (LPS), which was measured by studying CD69 and CD40 expression. Histograms are representative of n=4. The colors denote the following: red, unactivated; blue, activated, untreated; orange, activated, metformin; green, activated, BIG. **C**–**E**, Macrophages were treated with/without metformin (Met) or BIG (2 mmol/L) to determine the effect of these drugs on IL-6 (**C**), IL-12p40 (**D**), and IL-10 (**E**) production (n=4).

Our evidence that metformin inhibits inflammatory responses in hepatocytes independently of some metabolic actions prompted us to study inflammatory responses in extrahepatic tissues. Macrophages may undergo classical proinflammatory M1 activation in response to cues including lipopolysaccharide and interferon γ. However, in response to agents including IL-13 and IL-4, they may become M2 cells, which are generally thought of as having anti-inflammatory or tissue repair actions.^36^ We studied the effects of metformin and another drug biguanide (structurally this drug is the same as metformin except that it lacks the 2 methyl groups present in metformin), which we have found previously acts similar to metformin on hepatocytes.^6^ We investigated 3 aspects: macrophage differentiation, activation, and secretion of cytokines. We measured effects on bone marrow–derived macrophage (BMDM) differentiation into M1 and M2 macrophages, using expression of CD11c as a marker of M1 differentiation and CD206 as a measure of M2 differentiation. In addition, we investigated macrophage activation in response to lipopolysaccharide, which acts on the toll-like receptor TLR4, increasing expression of CD69 and CD40. There was no significant effect of the drugs on expression of any of these markers (Figure 4A and 4B). As in hepatocytes, metformin suppressed IL-1β gene expression in macrophages (Online Figure IIIA), but somewhat reminiscent of the effect of BI605906 on lipogenic genes, metformin increased expression of the other cytokines we had studied in hepatocytes (Online Figure IIIB–IIID). We did, however, observe further drug-induced reductions when we measured cytokine secretion, to investigate macrophage activity and differentiation more directly. The 3 cytokines we studied were inflammatory cytokines IL-12p40, IL-6, and the anti-inflammatory cytokine IL-10 in these TLR-triggered cells. Both drugs reduced IL-12p40 and IL-6 secretion but were without effect on IL-10 secretion (Figure 4C through 4E).

### Chronic Treatment of Hepatocytes With Low Doses of Metformin Triggers Anti-Inflammatory Signaling Responses Similar to Those Resulting From High-Dose Acute Treatment

**Figure 5.**
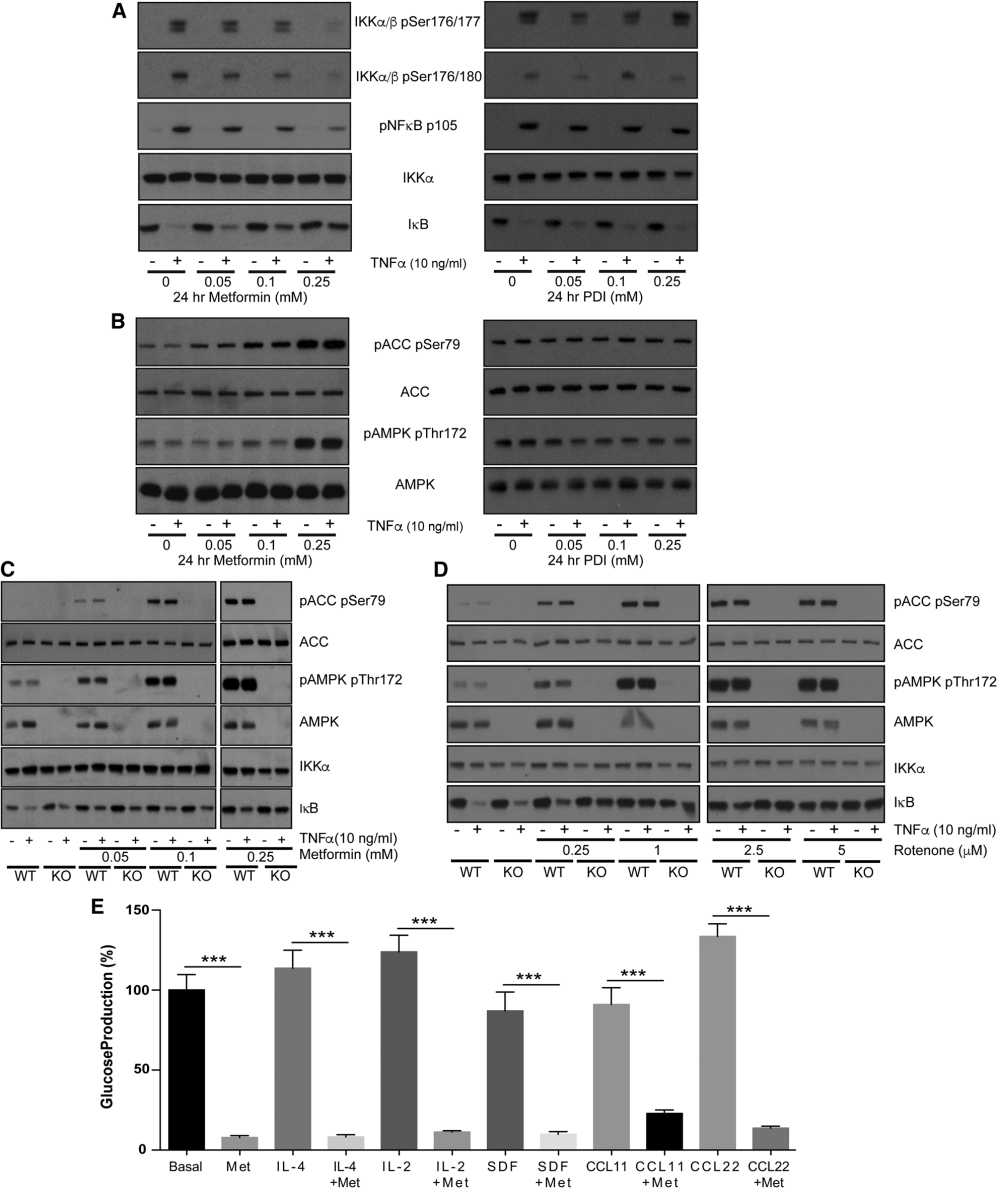
**Effect of long-term metformin treatment on nuclear factor**-κ**B (NF-κB) signaling responses in hepatocytes. A** and **B**, Primary hepatocytes were treated as in Figure 1 with metformin or propanediimidamide (PDI) at the doses indicated except that the treatment time was 24 h. For the last 15 min, cells were treated with 10 ng/mL tumor necrosis factor (TNF)-α. In addition to antibodies used elsewhere, phosphorylation of inhibitor of kappa B kinase (IKK)α/β was investigated using the phosphospecific antibodies indicated. After cell lysis, SDS-PAGE and immunoblotting were performed as in Figure 1. **C** and **D**, Hepatocytes from wild-type (WT) and AMPK double-knockout (KO) livers treated as in **A** or with doses of rotenone for 45 min before cell lysis, SDS-PAGE and immunoblotting. **E**, Primary hepatocytes were treated in the presence or absence of the agents shown. Cells were treated with/without metformin (2 mmol/L), C-C motif chemokine ligand (CCL)-11 (5 ng/mL), interleukin (IL)-2, IL-4, stromal cell–derived factor (SDF), and CCL22 (10 ng/mL) for 12 h, and glucose production was measured by GAGO (glucose [glucose oxidase]) assay as described in the Methods section of this article. ACC indicates acetyl-CoA carboxylase; AMPK, AMP-activated protein kinase; p-ACC, phospho–acetyl-CoA carboxylase; and pAMPK, phospho–AMP-activated protein kinase.

Plasma levels of metformin in the clinical setting are understood to be in the low micromolar range.^4,9^ Consequently, metformin-treated individuals may have lower intracellular concentrations of metformin than in our cell experiments, but the duration of exposure will be much longer. Discrepancies in effective concentrations of metformin likely occur because of the length of exposure, as the drug must accumulate in active mitochondria over several hours.^10,37^ In hepatocytes, long-term (24 hours) effects of the drug on NF-κB signaling occurred at concentrations close to the physiological range and this was unaffected by genotype (Figure 5A through 5C).

To provide more insight into the site of metformin action, we investigated signaling further upstream of IKK (Figure 5A). We found that TNF-α–induced phosphorylation of the upstream kinase site p176/177^38^ on IKKα/β was suppressed by metformin. In supporting studies, we found that propanediimidamide, a close structural analogue of metformin that we have found does not inhibit the mitochondria,^6^ does not inhibit IκB degradation, nor does it suppress phosphorylation of IKKα/β (Figure 5A). Consistent with the notion that NF-κB signaling can respond to mitochondrial inhibition independently of AMPK, we found that the complex I inhibitor rotenone prevented TNF-α–dependent IκB degradation in both genotypes (Figure 5D).

### Anti-Inflammatory Effects of Metformin in a DM Population Cohort

Next, we compared the effect of metformin and sulfonylureas on the NLR, a marker of inflammation that has recently been found to be a predictor of all-cause mortality and cardiac events.^31^ To test the hypothesis that metformin reduces inflammation using the GoDARTS diabetic cohort, we chose individuals prescribed metformin alone (without sulfonylurea or insulin) or sulfonylurea alone (without metformin or insulin), and for whom NLR measurements were available in the 120 days before first metformin/sulfonylurea prescription (the baseline measure) and 12 months after the first prescription (within a 8–16-month window). There were 498 people in metformin group and 172 in sulfonylurea group. Baseline characteristics of the 2 groups are shown in Table 1. Comparison of the 2 groups showed a significant effect of metformin exposure compared to sulfonylurea, with 12-month log-transformed NLR 0.09 lower in the metformin group (95% confidence interval [CI], 0.02–0.17; *P*=0.01), controlling for baseline values. This is equivalent to a 9% (95% CI, 2–15) lower geometric mean NLR. In addition, a logistic regression of 12-month NLR being lower than the baseline NLR gave an odds ratio of 1.83 (95% CI, 1.22–2.75; *P*=0.0034) for the metformin group compared with the sulfonylurea group (Tables 2 and 3). Body mass index (BMI) both nearest baseline and follow-up (4% missing) was not a significant variable (*P*=0.7), so was excluded. Inclusion of baseline HbA1c (19% missing) in the models resulted in similar effects. To examine the effect of metformin on high values on NLR, the models were rerun including only subjects with baseline NLR above the respective group median values (Tables 4 and 5). These showed a stronger metformin effect in the linear model, equivalent to a 15% (95% CI, 5–23) lower geometric mean NLR and an unchanged metformin effect for the logistic model, odds ratio of 1.91 (1.02–3.59). These results are summarized in Table 6.

**Table 1.**
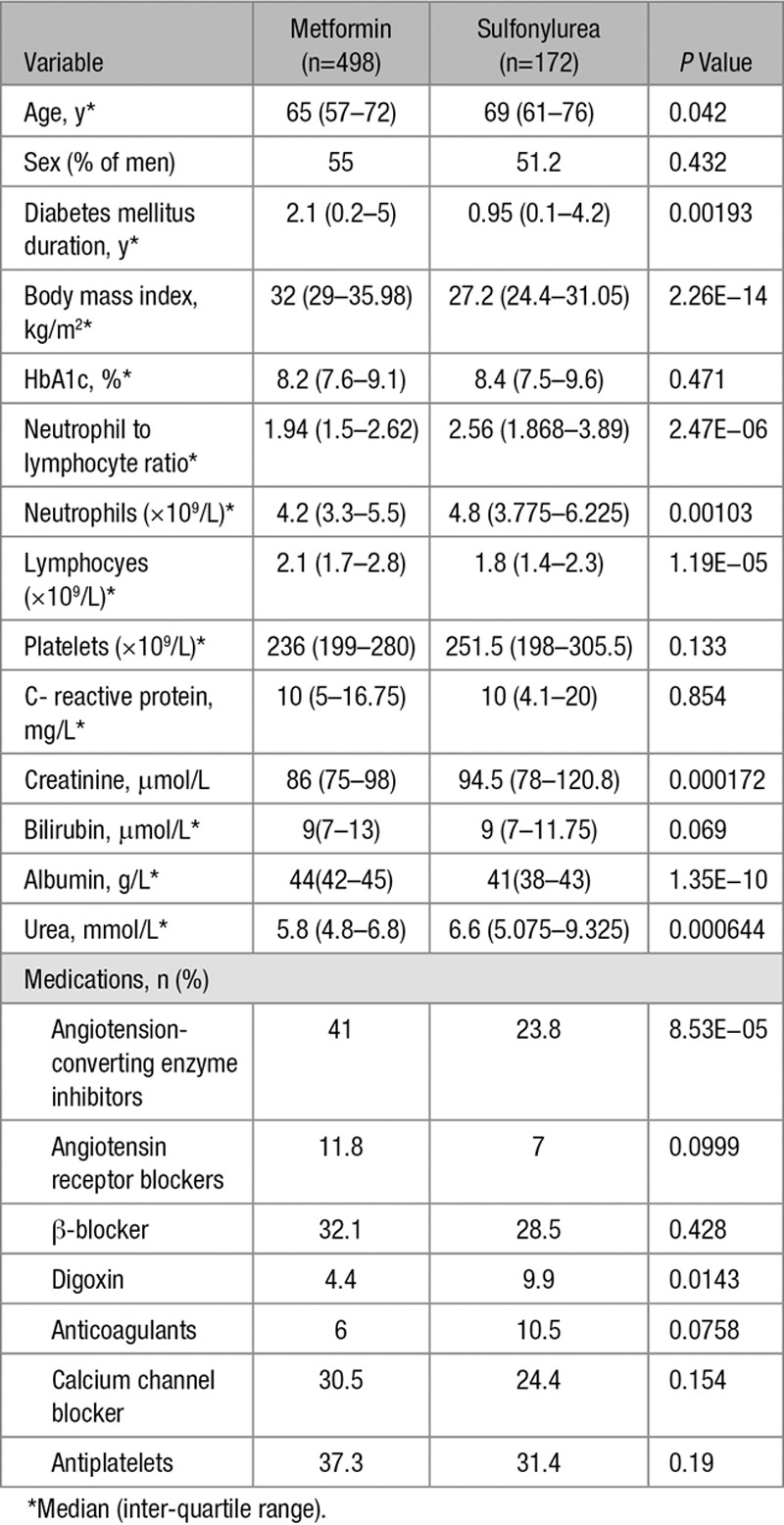
Baseline Measurements of GoDARTS (Genetics of Diabetes Audit and Research in Tayside Scotland) Diabetes Cohort

**Table 2.**
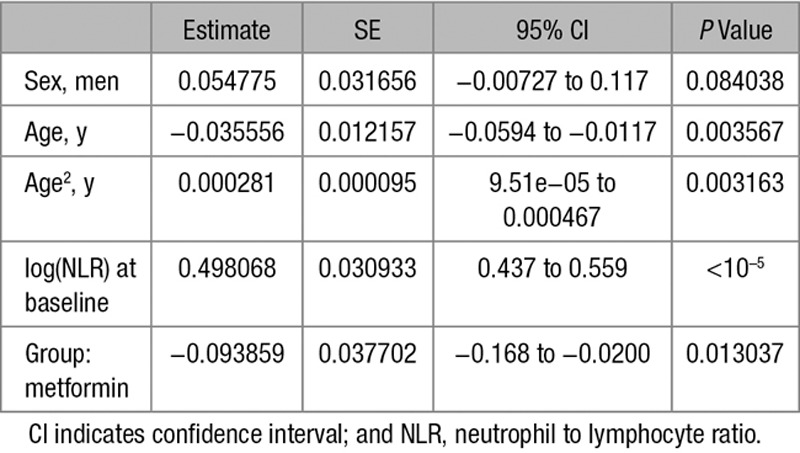
Regression Coefficients of Linear Model (log-NLR)

**Table 3.**
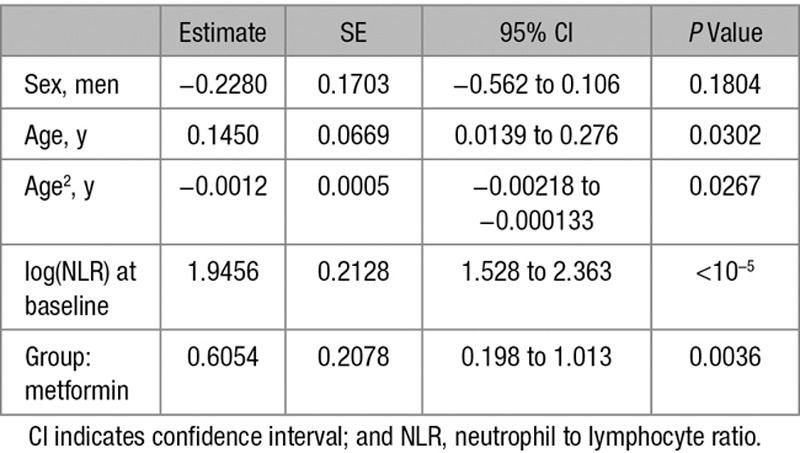
Regression Coefficients of Logistic Model (NLR_12<NLR_0)

**Table 4.**
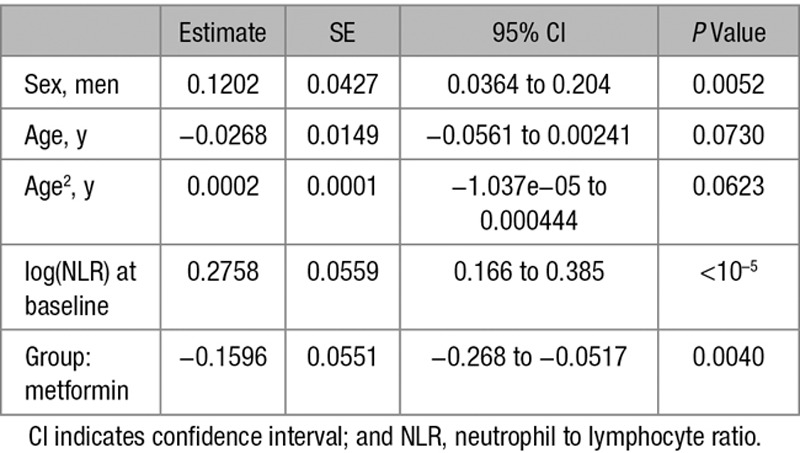
Regression Coefficients of Linear Model: Baseline NLR>Median

**Table 5.**
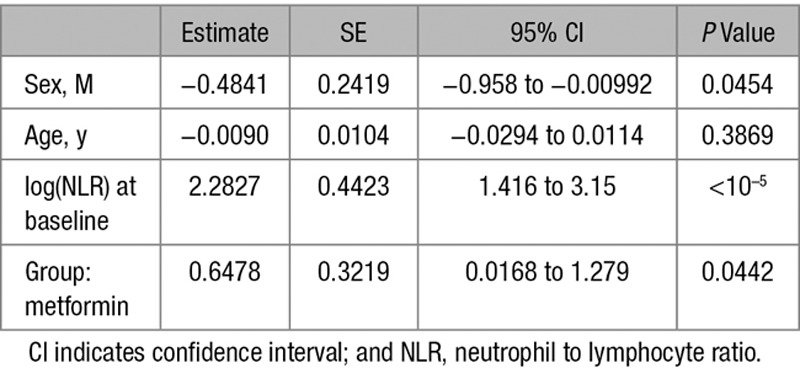
Regression Coefficients of Logistic Model: Baseline NLR>Median

**Table 6.**
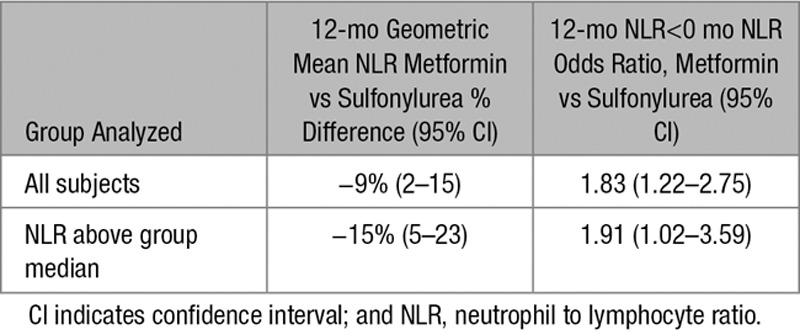
Summary of GoDARTS (Genetics of Diabetes Audit and Research in Tayside Scotland) Analyses, Comparing NLR in Metformin and Sulfonylurea Groups

To control for the different characteristics of the metformin and sulfonylurea groups, further analyses following propensity-score matching were performed. Nearest-neighbor one-to-one matching on DM duration, BMI, age, and angiotensin-converting enzyme exposure at baseline resulted in a reduced matched cohort of 318 (47% of original). Refitting the linear model using this cohort showed a similar effect of metformin exposure compared with sulfonylurea, with 12-month log-transformed NLR 0.10 lower in the metformin group (95% CI, 0.01–0.20; *P*=0.03). The logistic model for 12-month NLR lower than the baseline NLR gave an odds ratio of 1.53 for the metformin group compared with the sulfonylurea group; however, this effect was not statistically significant (95% CI, 0.93–2.52; *P*=0.096).

### Effect of Metformin on Inflammation in Nondiabetic Heart Failure

Given evidence that the anti-inflammatory effects of metformin may be dissociated from some metabolic responses in cells and from glycemic responses in DM, we further investigated the anti-inflammatory effects of metformin in a placebo-controlled clinical trial of metformin in a group of nondiabetic IR heart failure patients.^39^ In this study, compared with placebo, metformin significantly improved FIRI and resulted in a significant reduction in weight loss of 1.9 kg and BMI. Metformin treatment also reduced the prespecified secondary end point of the slope of the ratio of minute ventilation:carbon dioxide production.^39^ We analyzed plasma from 33 patients who took part in this study and performed multivariate ANOVA on all 40 cytokines with treatment (with and without metformin, 20 allocated to metformin and 13 to placebo) as the main factor and change in BMI as a covariate. This covariate analysis identified 5 cytokines that were significantly suppressed by metformin, after controlling for change in BMI (Table 7). Investigating these cytokines further, we performed Pearson correlations to identify cytokines significantly affected by metformin that correlated with a change in BMI. Among the 5 cytokines, correlations were observed for 2 of the 5 cytokines, CCL22 and CXCL12 (Table 8). Metformin improved insulin sensitivity as shown by significant reduction in FIRI (*t*=2.765, df=30.762; *P*<0.01) when an independent sample *t* test (equal variances not assumed) is performed; however, there was no significant correlation between change in FIRI and any change in the cytokines in the panel using a Pearson correlation. When a second correction for change in FIRI was applied, in addition to change in BMI, 4 of the 5 original cytokines remained significantly different with treatment (Table 7).

**Table 7.**
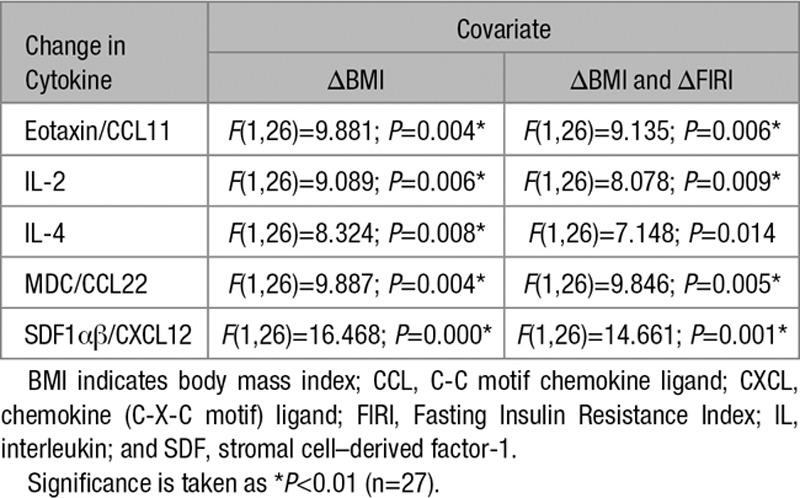
ANOVA of Heart Failure Cohort With Treatment (Without or With Metformin) as Main Factor With Covariate Analysis

**Table 8.**
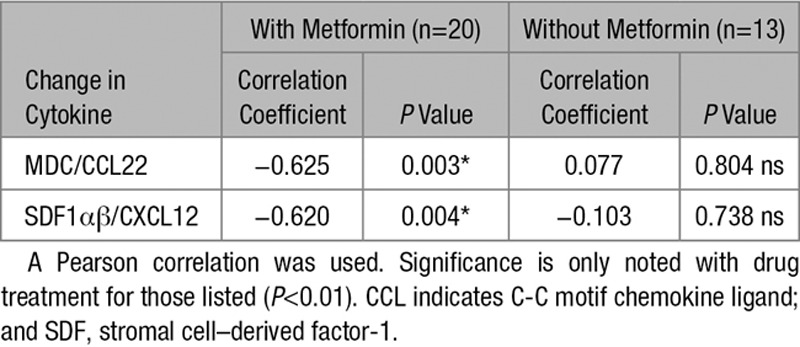
Correlation Between Change in Body Mass Index and the Cytokines That Were Significantly Affected by Metformin Treatment

Most of the cytokines suppressed by metformin in plasma were not measurable in hepatocytes or macrophages and for those that could be measured, metformin did not inhibit their expression in these cell types (Online Figure IV). Similar to our earlier studies, these cytokines had little, if any, effect on inducing glucose production in hepatocytes and metformin could still suppress this parameter in their presence (Figure 5E). All details of cytokine changes, metabolic, hemodynamic, and other parameters of these patients are described in Online Tables I and II.

## Discussion

We have used pharmacological and genetic approaches to isolate anti-inflammatory effects of metformin from those on glucose in cells, plasma, patient records, and in a placebo-controlled study. Initiating the study in hepatocytes, we separated signaling effects of metformin on the metabolic regulator AMPK from effects on inflammatory signaling. Although the AMPK activator AICAR induced similar effects to metformin on IκB degradation, AMPK was not required for these effects. In long-term treatment, effects of metformin on NF-κB signaling occurred at concentrations toward the physiological range, and in further studies, we found that propanediimidamide, a close structural analogue of metformin that does not inhibit the mitochondria,^6^ does not inhibit IκB degradation, nor does it increase phosphorylation of IKKα/β as is observed with metformin. Consistent with the possibility that NF-κB signaling can respond to mitochondrial inhibition independently of AMPK, we found that metformin and rotenone each prevented TNF-α–dependent IκB degradation in an AMPK-independent manner. Considering information from these pharmacological and genetic experiments, our data indicate that metformin acts upstream of IKKα/β through an AMPK-independent mechanism dependent on mitochondrial inhibition. This mechanism is fully consistent with our other observations that metformin does not directly inhibit IKK in vitro. These studies do not exclude the possibility of AMPK-dependent mechanisms contributing to anti-inflammatory actions of metformin in other ways. Effects of metformin on anti-inflammatory signaling pathways were separable from other metabolic responses to the drug. Inhibition of NF-κB signaling had little effect, for example, on glucose production or lipogenic gene expression, 2 key metabolic actions of metformin. Moreover, addition of cytokines suppressed by metformin in hepatocytes, plasma, or macrophages did not block the effect of the drug on glucose production. Taken together, these results define a dual action of metformin, with anti-inflammatory effects occurring alongside known antihyperglycemic and other metabolic effects. These 2 strands are both likely to be triggered by a mitochondrial target of the drug.

The evidence that metformin can suppress inflammatory signaling independently of some of its metabolic effects led us to investigate nonhepatic anti-inflammatory responses. Previous studies have suggested that inflammatory signaling on macrophages influences insulin sensitivity in other tissues. Loss of the lipopolysaccharide receptor TLR4, for example, confers some protection from insulin resistance following a high-fat diet.^40^ In addition, M2 macrophages dominate in adipose tissue in lean mice, whereas M1 macrophages accumulate in adipose tissue during obesity and are thought to contribute to systemic insulin resistance.^41^ At the level of gene expression, there were some differences between the effect of metformin in hepatocytes and macrophages although IL-1β was suppressed in both cell types. Studying cytokine secretion from macrophages, we found that metformin acted highly selectively to reduce proinflammatory cytokine secretion from activated macrophages, without affecting anti-inflammatory cytokine secretion and markers of macrophage differentiation and activation. This targeted mechanism may allow selective ablation of the ability of M1 macrophages to induce systemic insulin resistance in obesity. Taken together with the results in hepatocytes, this work suggests that metformin’s anti-inflammatory actions are likely to be qualitatively different from conventional NSAIDs.

We wished to establish whether the effects of metformin could be detected in humans and we started with a DM cohort. Investigating the GoDARTS patient database, we found evidence of metformin reducing subclinical inflammation as measured by NLR in patients. It is noteworthy that our findings support previous reports that metformin is capable of suppressing markers of inflammation such as high-sensitivity C-reactive protein in prediabetic individuals^42^ and TNF-α in IR individuals.^43^ NLR has recently been identified as a predictor of all-cause mortality and cardiovascular events,^31^ whereas previous studies demonstrated a substantial beneficial effect of metformin therapy on cardiovascular outcomes.^2,44,45^ Together, these results suggest that suppression of chronic inflammation by metformin might contribute to the difference in outcomes between these 2 treatment modalities.

Finally, given the evidence from cells that anti-inflammatory and metabolic effects of the drug can be separated, we studied a nondiabetic insulin-resistant heart failure cohort from a randomized controlled trial. Our research question was to determine whether metformin suppressed plasma cytokines. We observed a general trend of metformin treatment lowering cytokine concentrations. Correcting for change in BMI, 5 cytokines were significantly suppressed by metformin but only 2 of these, CCL22 and stromal cell–derived factor 1αβ, also correlated with change in BMI in follow-up analysis, suggesting that in individuals with established CVD, metformin exerts anti-inflammatory effects that are at least in part independent of BMI. Four of the 5 cytokines remained significant after additional correction for FIRI, and there was no significant correlation between change in FIRI and any of the cytokines in the panel using a Pearson correlation, even though metformin did reduce FIRI. Together these data strongly suggest that metformin has effects above and beyond the known effects on BMI and insulin sensitivity. The identity of these 5 cytokines signpost ways in which anti-inflammatory effects of metformin could exert DM-independent therapeutic effects in CVD. One earlier cohort study, for example, found that a Thr/Ala substitution in the CCL11 gene increases risk of myocardial infarction independently of BMI and DM.^46^ Blockade of CCL11 can suppress aspects of age-related cellular dysfunction,^47^ and it is possible that observed effects of metformin on mammalian longevity,^48,49^ where suppression of NF-κB is also observed,^49^ may owe at least in part to suppression of this cytokine. The other cytokines stromal cell–derived factor 1αβ, IL-2, IL-4, and CCL22 are each implicated in resolution of pancreatic β cell inflammation^50–53^ and stromal cell–derived factor 1αβ, IL-2, and IL-4 are additionally upregulated in plasma from type 2 DM individuals.^54,55^ Further work will be required to determine how the effects on macrophages and hepatocytes that we have measured contribute to the changes in plasma cytokines observed. Changes in other inflammatory cell types, particularly neutrophils given the change in NLR, or in cell–cell interactions, may need to be taken into account. Altogether our results are consistent with metformin exerting a potentially cardioprotective anti-inflammatory effect in patients with CVD, suppressing both age and metabolic inflammatory stress markers, independently of effects on BMI, insulin sensitivity, and without the onset of frank DM.

We recognize the limitations that are inherent in retrospective, nonrandomized, observational cohort data. It was impossible to account for all possible confounding influences that may have biased the observed differences between the groups considered. For example, the BMI of the 2 groups is different, consistent with historical prescribing patterns (Tables 7 and 8). We have sought to minimize these as far as practicable by 3 different sensitivity analyses. First, by using a multivariate model adjusting for potential confounders; second, by performing a propensity score–matched analysis; and third, we detected an anti-inflammatory signal in a randomized, double-blinded, placebo-controlled trial, providing definitive evidence of anti-inflammatory effects of metformin in this group of patients. The propensity score–matched analysis has been shown to eliminate as much as 90% of treatment bias in observational studies.^56^ Because of the small size of the clinical trial, this proof-of-concept study was designed and powered only to investigate the study-specific end point of peak oxygen uptake in patients with heart failure and not on clinical outcome. However, we have previously shown in a large population-based cohort study that patients with DM and heart failure who were treated with metformin alone or in combination with sulfonylureas were at significantly lower risk of all-cause mortality during 1 year and long-term follow-up than those who were treated with sulfonylurea alone.^44^ Our findings on metformin and inflammation will now similarly need to be confirmed in other patient cohorts.

In summary, cross-species evidence from cells, plasma, patient records, and a randomized placebo-controlled study strongly suggest that anti-inflammatory effects should be investigated further as a potentially important aspect of metformin’s clinical pharmacology that may particularly accelerate investigation of their utility in nondiabetic cohorts. There is overwhelming evidence that inflammation contributes to the development of CVD^24^ but counterbalancing this is evidence from meta-analysis of randomized control trials that existing NSAIDs tend to exacerbate risk of CVD.^57^ If inflammation is to be targeted successfully in CVD, new treatment paradigms will need to be established. It is likely, for example, that agents targeting only selected aspects of inflammation will need to be identified. Our work identifying discrete anti-inflammatory effects of metformin on cell signaling and plasma parameters independently of DM supports ongoing and prospective investigation into repurposing metformin in a broader spectrum of patients with CVD.

## Acknowledgments

We thank Dr Kashyap Patel (Exeter) for demonstrating hepatocyte extraction. In addition, Dr Andy Cassidy and Dr Ritu Sharma (both Dundee) assisted set-up of real-time polymerase chain reaction.

## Sources of Funding

G. Rena acknowledges funding from MRC (MR/K012924/1) and the Diabetes UK RW and JM Collins studentship, supporting C. Forteath (12/0004625). S.C. Fagerholm acknowledges funding from the Academy of Finland and Biocentrum Helsinki. M. Foretz acknowledges funding from the Région Ile de France-CORDDIM and by the Société Francophone du Diabète. C.C. Lang acknowledges support from the British Heart Foundation (grant number PG/06/143/21897 and PG/14/4/30539). A.K.F. Wong acknowledges support from the British Heart Foundation (grant number PG/06/143/21897). M. Mohan. acknowledges fellowship support from the British Heart Foundation (grant number PG/14/4/30539). V.L. Morrison was supported by the Ella and Georg Ehrnrooth foundation and D.J.K. Balfour acknowledges funding from Alzheimer’s Research UK, grant number ART-EXT-2010–2. C. Beall is an RD Lawrence Fellow (Diabetes UK grant number: 13/00004647).

## Disclosures

None.

## Supplementary Material

## Novelty and Significance

### What Is Known?

- Observational studies have repeatedly shown benefit of metformin in reducing incidence of CVD events in DM.
- Inflammation is understood to contribute to CVD cause, but it has been difficult to harness anti-inflammatory effects for CVD therapy.
