# Supplementary material for: Anti-Inflammatory Effects of Metformin Irrespective of Diabetes Status
Source: Circ Res. 2016 Aug 18;119(5):652–65. doi: 10.1161/CIRCRESAHA.116.308445 (PMC4990459; doi:10.1161/CIRCRESAHA.116.308445)
Supplement: Supplementary file 1 [file res-119-652-s001.pdf]

Circulation Research

From: Ritu Sharma (Staff) <R.S.Sharma@dundee.ac.uk>
Sent: Monday, July 11, 2016 5:24 AM
To: circulation.research@circresearch.com
Cc: Amy Cameron (Staff)
Subject: CIRCRES/2016/308445R2 :Permission of Acknowledgement

Hi

I write here to confirm that I am happy with my name cited in acknowledgements for assisting in setting up of RT-PCR, for the manuscript in reference:
CIRCRES/2016/308445R2: Anti-inflammatory effects of metformin irrespective of diabetes status.

Please let me know if you need any further information.

Many thanks & kind regards
Ritu

Dr Ritu S Sharma
Prof. John Hayes Lab
Division of Cancer Research,
School of Medicine
Jacqui Wood Cancer Centre, University of Dundee
James Arrott Drive, Ninewells Hospital And Medical School
Dundee DD1 9SY Scotland

From: Amy Cameron (Staff)
Sent: 07 July 2016 09:57
To: Andrew Cassidy (Staff); Ritu Sharma (Staff)
Cc: Graham Rena (Staff)
Subject: Permission of Acknowledgement

Dear Ritu and Andy

Our recently submitted manuscript to Circulation Research is on the cusp of being accepted. Prior to this acceptance the journal has requested that anyone we have acknowledged by name must cite their permission to be acknowledged directly to the journal. I would really appreciate if you could send an email to circulation.research@circresearch.com stating you are happy with this. The reference for the manuscript is CIRCRES/2016/308445R2: Anti-inflammatory effects of metformin irrespective of diabetes status. You have been acknowledged as follows: In addition, Dr Andy Cassidy and Dr Ritu Sharma (both Dundee) assisted set-up of RT-PCR.

Thanks.

Amy

Dr Amy Cameron

Circulation Research

From: Andrew Cassidy (Staff) <a.j.cassidy@dundee.ac.uk>
Sent: Thursday, July 07, 2016 7:59 AM
To: circulation.research@circresearch.com
Cc: Amy Cameron (Staff)
Subject: Acknowledgement

Dear Circulation Research,

I am happy with this acknowledgement, thank you.

The reference for the manuscript is CIRCRES/2016/308445R2: Anti-inflammatory effects of metformin irrespective of diabetes status. You have been acknowledged as follows: In addition, Dr Andy Cassidy and Dr Ritu Sharma (both Dundee) assisted set-up of RT-PCR.

Kindest regards

Dr Andrew Cassidy
The Tayside Centre for Genomic Analysis
University of Dundee
School of Medicine, Level 6
Ninewells Hospital and Medical School
DD1 9SY
Phone (Office): 01382 383268
Phone (Mobile) 07794578063
E-Mail: a.j.cassidy@dundee.ac.uk
Web: http://www.tcga.org.uk

The University of Dundee is a registered Scottish Charity, No: SC015096

Circulation Research

From: Patel, Kashyap <K.A.Patel@exeter.ac.uk>
Sent: Thursday, July 07, 2016 7:30 AM
To: circulation.research@circresearch.com
Cc: g.rena@dundee.ac.uk
Subject: Fwd: Permission of Acknowledgement

Dear Sir / Madam

I am happy for my acknowledgement in the following manuscript

CIRCRES/2016/308445R2: Anti-inflammatory effects of metformin irrespective of diabetes status

Best wishes

Kash

Sent from my iPhone

Begin forwarded message:

From: "Amy Cameron (Staff)" <A.X.Cameron@dundee.ac.uk>
Date: 7 July 2016 at 04:48:03 GMT-4
To: "'k.a.patel@exeter.ac.uk'" <k.a.patel@exeter.ac.uk>
Cc: "Graham Rena (Staff)" <g.rena@dundee.ac.uk>
Subject: Permission of Acknowledgement

Dear Kash

Our recently submitted manuscript to Circulation Research is on the cusp of being accepted. Prior to this acceptance the journal has requested that anyone we have acknowledged by name must cite their permission to be acknowledged directly to the journal. I would really appreciate if you could send an email to circulation.research@circresearch.com stating you are happy with this. The reference for the manuscript is CIRCRES/2016/308445R2: Anti-inflammatory effects of metformin irrespective of diabetes status. You have been acknowledged as follows: We thank Dr Kashyap Patel (Exeter) for demonstrating hepatocyte extraction.

Thanks.

Amy

Dr Amy Cameron
Division of Molecular & Clinical Medicine (Level 5)
Mailbox 12
School of Medicine
Ninewells Hospital & Medical School
University of Dundee
