# Supplementary material for: Anti-Inflammatory Effects of Metformin Irrespective of Diabetes Status
Source: Circ Res. 2016 Aug 18;119(5):652–65. doi: 10.1161/CIRCRESAHA.116.308445 (PMC4990459; doi:10.1161/CIRCRESAHA.116.308445)

Supplementary Materials

Expanded Methods

I. Animal and Cell Studies

Metformin and rapamycin came from Calbiochem, AICAR and A769662 (Tocris), TNFα (e-bioscience), recombinant CINC1/CXCL1, CCL-11, IL-2, IL-4, SDF and CCL22 (R&D systems), mouse IL-6 (Sigma) and recombinant mouse IL-1β (Life Technologies). The phospho-acetyl-CoA carboxylase (ACC) Ser79 antibody was a generous gift from the DSTT (University of Dundee). The total ACC (Cat. number 3662), total AMPKα (2603), phospho-AMPKα Thr172 (2535), total S6 (2217), phospho-S6 Ser240/244 (2215), total p70 S6 kinase (2708), phospho-p70 S6 kinase Thr389 (9205), phospho-Raptor Ser 792 (2083), phospho IKKα/β Ser176/177 (2078), IKKα/β Ser176/180 (2697), total IκB, pNF-κB, total IKKα and total IKKβ (NF-κB sampler kit 9936) antibodies were from CST. Anti-sheep HRP (31480) and anti-rabbit HRP (31460) both came from Thermo and anti-mouse HRP was from Calbiochem (JA1200). BI605906 was a generous gift from Prof Sir Philip Cohen.

Animal Care

C57BL/6 female mice (Charles River, 8-41 weeks) were maintained under a 12 hours:12hours light:dark cycle (holding room lights on at 06:00; off at 18:00) at 22±1°C and 50% humidity. Mice had *ad libitum* access to standard chow diet (7.5% fat, 75% carbohydrate and 17.5% protein by energy (RM1 diet; Special Diet Services) and water. All animal care protocols and procedures were performed in accordance with current regulations.

AMPKα1α2-null (AMPK KO) mice were maintained under a 12-hour light/12-hour dark cycle with free access to water and standard mouse diet (in terms of energy: 65% carbohydrate, 11% fat, 24% protein). These AMPK catalytic subunit deficient mice were generated as previously described (1). All procedures were performed in accordance with the principles and guidelines established in the *European Convention for the Protection of Vertebrate Animals Used for Experimental and Other Scientific Purposes* (Council of Europe, ETS no. 123, 1991).

Hepatocyte Extraction

Mice were killed by cervical dislocation following guidelines set out by the Animals (Scientific Procedures) Act 1986. An incision into the abdomen was followed by dissection of the skin, abdominal cavity and diaphragm to expose the liver, kidney, inferior vena cava and portal vein. The superior vena cava was clamped to isolate the hepatic system and the inferior vena cava was cannulated just above the kidney with a 25G butterfly needle and clamped in place. Immediately after cannulation, the portal vein was cut. Successful cannulation was determined when the liver cleared quickly of blood and became pale throughout. The liver was perfused with 50ml pre-warmed perfusion buffer (137mM NaCl, 7mM KCl, 0.7mM Na_2HPO_4, 10mM HEPES pH 7.65 filter sterilized (0.2 µm) with 0.1% EDTA 0.5M pH 8 added just prior to use) at a rate of 5ml/min. After 10 min, the liver was perfused with 50ml digestion buffer (perfusion buffer without EDTA with 5.1mM $CaCl_2$ and 20mg collagenase (from Clostridium histolyticum type IV, Sigma)); added at a rate of 5ml/min. After digestion, the liver was excised from the abdominal cavity and transferred to a cell culture hood in a 10cm dish. The liver was resuspended in 10ml of plating media (440ml M199 + Glutamax (1x), Invitrogen; 5ml Pen/Strep (100x), Invitrogen; 6.7ml BSA (7.5%), Invitrogen; 50ml FBS (foetal bovine serum); 7.7µl Insulin Actrapid (100U/ml), Novo Nordisk; 100µl T3 (thyroid hormone 1mM stock), Sigma; 25µl Dexamethasone (10mM stock), Merck) and the hepatocytes isolated by gently agitating the liver. Cells were filtered through a 100 µm cell strainer and this process was repeated 4 more times until a final volume of 50ml was obtained. Hepatocytes were pelleted by centrifugation at 400 rpm for 5 min using no acceleration or braking. The supernatant was discarded and the cell pellet resuspended in 25ml plating media by gentle inversion. Cell viability was determined by 0.04% Trypan blue staining and the cell number determined using a haemocytometer. Cell viability of >90% was required for experimental use.

Cell Culture and Lysis for Immunoblotting

All cells were maintained in an incubator at 37ºC and 5% CO_2. For lysate and RT-PCR experiments, primary mouse hepatocytes were plated in 6-well plates (2.5×10^5 cells/well in 2ml media) while for glucose assay experiments, primary mouse hepatocytes were plated in 12-well plates (1.25×10^5 cells/well in 1ml media). After 4

hours, plating media was removed, cells were washed with warmed PBS and overnight media (500ml M199 + Glutamax (1x); 5ml Pen/Strep (100x); 25μl Dexamethasone (10mM stock)) was added at 2ml per well. Cells were incubated overnight and experiments were performed the following day.

BMDMs were grown from mouse bone marrow in RPMI 1640 medium supplemented with 10% FBS (Life Technologies) and 10ng/ml M-CSF (R&D systems). Cells were given fresh medium and growth factor on day 3 of culture. On day 6, BMDM cultures were supplemented with 100ng/ml IFNγ (for M1 differentiation; R&D systems), 20ng/ml IL-4 (for M2 differentiation; R&D systems), or 100ng/ml LPS (for activation; premium grade from Sigma, expected to activate TLR2 and TLR4) in the presence or absence of drug treatments for the final 24h.

Prior to SDS-PAGE, cells were lysed by scraping into buffer A: (50mM Tris acetate pH7.5, 1% (w/v) Triton X100, 1mM EDTA, 1mM EGTA, 0.27M sucrose, 50mM NaF, 1mM sodium orthovanadate, 10mM β-glycerophosphate, 5mM sodium pyrophosphate, 1mM benzamidine, 0.2mM phenylmethylsulfonyl fluoride (PMSF) and 0.1% (v/v) β-mercaptoethanol) then prepared for SDS-PAGE as follows. The lysates were centrifuged at 13 000 g for 15 min, and the supernatants were removed. Protein concentration was determined by Bradford assay (Bio-Rad). The supernatant was loaded in equal amounts of protein and subjected to 4-20% gradient SDS-PAGE and subsequently were transferred to nitrocellulose membranes. Primary antibody incubations were performed at dilutions recommended by the manufacturer or determined by us in 5% milk TBS-T. All incubations were done at 4°C, overnight after a 1 hr block in 5% milk TBS-T. The secondary antibody was used at 1:5000 dilution for 1 hr at room temperature. Proteins were visualised using the enhanced chemiluminescence (ECL) system (Amersham) onto X-ray film (Kodak). Immunoblot densitometry for each antibody was performed with Image Studio Lite version 5.2 (LI-COR). Each blot is representative of experiments carried out at least three times.

Glucose Assay

Treatment of cells for hepatocyte glucose production was performed using primary mouse hepatocytes plated in 12-well plates (1.25 x 10^5 cells/well in 1ml media).

Glucose production was determined after a 12 hour incubation period in 750µl glucose-free DMEM (11966; Life Technologies) supplemented with 1% Pen/Strep, lactate (Sigma)/pyruvate (Life Technologies) (10:1 mM) and 100nM dexamethasone (dex; Merck) with or without drugs/cytokines under investigation. At the end of the incubation period of 12 hours, 500µl of medium was collected and glucose concentration determined by GAGO assay (GAGO-20; Sigma) by a modified protocol scaled down to a 96-well plate format. 50µl of sample medium followed by 100µl assay reagent was added to each well with no time delay. Following incubation at 37°C for 30 minutes, 100µl 12N H_2SO_4 was added to each well and mixed using a multi-well pipette. Absorbance was measured at 540 nm. Each column consists of data from at least 12 wells of cells, six each from two mice.

RT-PCR

Primary mouse hepatocytes were incubated for 8 hours in 1ml glucose-free DMEM (11966; Life Technologies) supplemented with 1% pen/strep, lactate (Sigma)/pyruvate (Life Technologies) (10:1 mM) and 100nM dexamethasone (dex; Merck) with or without drugs under investigation. After this incubation period, media was removed and cells were washed once with warmed PBS. 350µl of Buffer RLT from the Rneasy MINI KIT (Qiagen) plus 10% β-mercaptoethanol was added to each well. Plates were then placed on ice for 10 min, followed by a cell harvest and samples were snap-frozen immediately in LN_2. Total RNA was extracted using QIAshredder (Qiagen) and Rneasy MINI KIT (Qiagen) as per the manufacturer's instructions. cDNA was synthesized from 1µg RNA using RQ1 Rnase-Free Dnase kit (Promega) and ImProm-II Reverse Transcription System (Promega). cDNA was diluted in nuclease-free water 1:10 prior to use.

Nucleospin RNA II Total RNA isolation kit (Macherey-Nagel) was used to isolate RNA from macrophages. cDNA was synthesized from 0.5µg RNA using High Capacity cDNA Reverse Transcription Kit (4368814, Thermo Fisher Scientific). cDNA was diluted in nuclease-free water 1:2 prior to use.

Real-time PCR was carried out using the 7900HT Fast Real-Time PCR System (Applied Biosystems) using TaqMan 2x Universal PCR Master Mix (Applied Biosystems) and primer/probes mixes as stated (Applied Biosystems). Primer sets

used were: IL-6 Mm00446190_ml; CXCL1 Mm04207460_m1; 18S Hs03003631_g1; IL-1β Mm00434228_m1; CXCL2 Mm00436450_m1; PPARγ Mm01184322_m1; FASN Mm00662319_m1; CCL22 Mm00436439_ml; CXCL12 Mm00445553_ml; TBP Mm01277042_m1 and SREBP-1c Mm00550338_m1. Cycling conditions were: 50°C for 2 min, 95°C for 10 min, followed by 40 cycles of 95°C for 15 s and 60°C for 1 min. Expression is expressed relative to 18s mRNA for hepatocytes and TBP for macrophages (Applied Biosystems) using the $2^{-\Delta\Delta Ct}$ method. Control samples were set at a value of 100% and results for all experimental samples were graphed as relative expression compared to control. Each column is composed of data from at least three separate experiments.

BMDM studies

BMDMs were harvested from culture plates using 4mM EDTA in PBS for 10min at 37°C. Cells were washed in flow cytometry buffer (PBS with 2% FBS and 1mM EDTA) and stained using the following antibodies (all BD Bioscience unless stated): F4/80 (BM8; e-bioscience), CD11c (HL3), CD206 (C068C2; Biolegend), CD69 (H1.2F3) and CD40 (3/23). Fc block (4.4G2) was included in all stains. Data were acquired on a LSR II flow cytometer (Becton Dickinson) and analysed using FlowJo software (TreeStar). BMDM culture supernatants were collected after 24 hours treatment with the differentiation or activation conditions. Levels of cytokines were quantified by standard sandwich ELISA using paired antibody kits (e-bioscience), according to the manufacturer's instructions.

Statistical Analyses

Results in bar graphs are expressed as mean ± SEM. Comparisons between groups were made by one-way ANOVA with Dunnett's or Tukey post-hoc test using Prism. Differences were considered statistically significant if *P* was less than 0.05. *** denotes $p<0.001$; ** denotes $p<0.01$ and * denotes $p<0.05$. For studies on the plasma, statistical analyses of data were performed using SPSS 14.1. ANOVA and Pearson correlation coefficients were calculated.

II. Validation in Clinical Patients

Population Cohort Study: Diabetes Patient Metformin Exposure and Neutrophil-to-Lymphocyte Ratio (NLR).

Sample Ascertainment

Patients were ascertained from the Diabetes Audit and Research in Tayside Scotland (DARTS) study, which has previously been described in detail (2). In brief, all the participants were linked through to the Health Informatics Centre Database to retrieve validated prescribing information, clinical information system, all haematological and biochemistry data and the Scottish Care Information–Diabetes Collaboration (SCI-DC) (REF) that provide additional clinical phenotypic data back to 1992. Prospective longitudinal data were also collected on these patients. The study was approved by the Tayside Regional Ethics Committee, and informed consent was obtained from all subjects since 1997 to DNA and serum collection as part of the Wellcome Trust United Kingdom Type 2 Diabetes Case Control Collection. Over 17,000 subjects have participated in this Genetics of DARTS (Go-DARTS) study till date, of whom over 9,000 have diabetes.

Hematological Measurements.

We analysed the electronically linked records of routine laboratory investigations of all the participants from the regional biochemistry and hematological database. The total and differential leucocyte counts (including the neutrophils and lymphocytes) were determined from peripheral venous blood samples using an automated Siemens' high-volume hematology analyzer, the ADVIA® 2120i System (peroxidase method) (3). NLR was calculated as the ratio between (percentage of) neutrophils and total lymphocyte counts in the study subjects.

Statistical Analysis

For the population cohort study, characteristics of patients with or without metformin therapy were compared by the chi-square test for categorical variables and by the t test or Mann-Whitney U test for continuous variables as appropriate. The effect of metformin therapy on NLR were examined together with the significant differences found at baseline using linear and logistic regression analysis. The following covariates were included: age, sex, HbA1c, BMI, duration of followup, prior

hospitalisation for COPD, Atrial Fibrilation or Hypertension, and baseline NLR. To minimize confounding influences, we performed two different sensitivity analysis. First, by using a multivariate model adjusting for potential confounders; second we determined a propensity score using a logistic regression model to control for the different characteristics of the metformin and sulfonylurea groups. A P-value of <0.05 was considered significant and all statistical analysis for this cohort study were performed using R for windows (v3.2.0).

Randomised Placebo Controlled Study

The effect of metformin on plasma inflammatory cytokines were further investigated in a subset of chronic heart failure (CHF) patients who had participated in a double-blind, placebo-controlled study of metformin, which has previously been described in detail (4). In brief, this study was designed and powered to evaluate the impact of metformin on IR and its effects on exercise capacity in non-diabetic IR patients with CHF. Every patient who participated in this study, provided written informed consent prior to participation in this study, which was approved by the East of Scotland Research Ethics Service (www.clinicaltrials.gov: NCT00473876). In this study we had shown that metformin treatment significantly improved IR but had no significant effect on the primary endpoint of exercise capacity, as measured by peak VO_2. However, metformin treatment did result in a significant improvement in the minute ventilation − carbon dioxide production relationship (VE/VCO_2 slope), a pre-specified secondary endpoint of this proof of concept study which is of prognostic significance in patients with CHF, and in some studies, it has outperformed peak VO_2 (5).

Cytokine Assay

We analysed plasma from 33 non-diabetic insulin resistant heart failure patients who took part in a placebo controlled clinical trial of metformin (4). The plasma was analysed using the Bio-Plex Pro Human Chemokine 40-Plex Panel (171-AK99MR2, Bio-Rad). The assay was performed following the manufacturer's instructions using the Bio-Plex 200 system (Bio-Rad). Freeze-thaw cycling of samples was avoided to prevent cytokine degradation and they were diluted 1:4 (12.5μl of plasma) for the assay.

References

(1) Foretz M, Hébrard S, Leclerc J, Zarrinpashneh E, Soty M, Mithieux G, Sakamoto K, Andreelli F, Viollet B: Metformin inhibits hepatic gluconeogenesis in mice independently of the LKB1/AMPK pathway via a decrease in hepatic energy state*. J Clin Invest* 2010;120:2355-2369.
(2) Morris AD, Boyle DI, MacAlpine R, Emslie-Smith A, Jung RT, Newton RW, MacDonald TM: The diabetes audit and research in Tayside Scotland (DARTS) study: electronic record linkage to create a diabetes register. DARTS/MEMO Collaboration. *BMJ* 1997;315:524-528
(3) ADVIA 2120i Hematology System with Autoslide. SIEMENS Healthineers website. http://www.healthcare.siemens.com/hematology/systems/advia-2120-hematology-system-with-autoslide. Updated September 9, 2015. Accessed July 7, 2016.
(4) Wong AKF, Symon R, Alzadjali MA, Ang DSC, Ogston S, Choy AM, Petrie JR, Struthers AD, Lang CC: The effect of metformin on insulin resistance and exercise parameters in patients with heart failure. *European Journal of Heart Failure* 2012;14:1303-1310
(5) Arena R, Myers J, Aslam SS, Varughese EB, Peberdy MA. Peak VO2 and VE/VCO2 slope in patients with heart failure: a prognostic comparison. *Am Heart J* 2004; 147: 354–360.

Supplementary table I. Cytokine measurements

	Baseline			
	Placebo (n=13)		Metformin (n=20)	
Cytokine (pg/ml)	Average	SD	Average	SD
CCL21/6Ckine	5454.29	2067.61	5890.92	1853.73
BCA-1/CXCL13	28.52	9.54	35.02	13.22
CTACK/CCL27	1723.32	583.41	1973.63	815.37
ENA-78/CXCL5	642.91	213	781.07	321.26
Eotaxin/CCL11	51.4	13.67	60.8	17.69
Eotaxin-2/CCL24	685.11	518.81	640.79	402.68
Eotaxin-3/CCL26	56.27	18.48	74.93	60.97
Fractalkine/CX3CL1	172.05	61.95	237.93	93.3
GCP-2/CXCL6	20.34	8.37	26.27	14.34
GM-CSF	128.91	50.39	137.67	52.51
Gro-α/CXCL1	348.61	83.78	336.15	76.27
Gro-β/CXCL2	153.61	74.54	235.74	178.19
I-309/CCL1	83.83	17.2	92.43	18.49
IFNγ	64.35	20.88	75.98	27.42
IL-1β	7.53	3.66	9.33	3.84
IL-2	15.96	5.21	18.87	6.48
IL-4	32.19	7.45	33.4	9.48
IL-6	14.14	3.89	17.81	8.58
IL-8/CXCL8	14.19	4.16	16.9	4.72
IL-10	46.74	21.99	54.49	22.46
IL-16	329.73	120.54	364.8	130.41
IP-10/CXCL10	173.62	67.61	206.57	114.88
I-TAC/CXCL11	18.02	6.86	23.33	7.83
MCP-1/CCL2	69.92	33.76	86.03	52.74
MCP-2/CCL8	56.84	21.19	70.44	38.9
MCP-3/CCL7	126.6	35.08	146.64	53.26
MCP-4/CCL13	77.59	46.32	69.26	39.3
MDC/CCL22	1014.65	290	1151.86	505.54
MIF	6303.03	5822.99	5681.16	3323.86
MIG/CXCL9	296.66	136.95	404.71	277.22
MIP-1α/CCL3	10.5	2.02	12.22	3.69
MIP-1δ/CCL15	7487.28	3978.97	7716.92	4125.49
MIP-3α/CCL20	34.9	79.52	37.84	43.88
MIP-3β/CCL19	353.04	252.2	463.93	308.82
MPIF-1/CCL23	446.78	221.81	494.74	211.13
SCYB16/CXCL16	503.06	216.96	567.01	228.79
SDF-1α+β/CXCL12	1257.91	423.96	1487.04	500.84

TARC/CCL17	113.29	81.29	138.35	94.4		
TECK/CCL25	759.25	248.21	896.76	327.17		
TNFα	46.33	10.03	51.18	15.66		
	Change after 4 months metformin treatment					
	Placebo (n=13)		Metformin (n=20)		BMI (p value)	BMI & FIRI (p value)
**Cytokine (pg/ml)**	**Average**	**SD**	**Average**	**SD**		
CCL21/6Ckine	99.20	756.47	55.16	2595.81	0.857	0.8
BCA-1/CXCL13	3.92	7.29	2.34	14.28	0.036	0.029
CTACK/CCL27	54.09	401.40	-128.67	902.78	0.022	0.032
ENA-78/CXCL5	29.79	148.93	-60.30	375.81	0.02	0.025
Eotaxin/CCL11	5.18	8.94	-0.36	17.00	0.004 *	0.006 *
Eotaxin-2/CCL24	32.73	258.03	-21.23	210.01	0.309	0.356
Eotaxin-3/CCL26	6.54	13.67	-11.50	54.48	0.038	0.053
Fractalkine/CX3CL1	0.93	56.10	-25.89	107.44	0.085	0.081
GCP-2/CXCL6	0.91	6.29	1.05	13.11	0.169	0.216
GM-CSF	1.52	34.36	3.15	55.77	0.051	0.05
Gro-α/CXCL1	11.01	54.90	5.89	80.00	0.101	0.106
Gro-β/CXCL2	32.80	56.92	9.41	120.75	0.317	0.365
I-309/CCL1	6.16	10.89	-0.69	17.79	0.011	0.014
IFNγ	6.28	16.04	-1.85	29.38	0.019	0.025
IL-1β	0.16	2.90	-0.96	4.15	0.116	0.098
IL-2	1.47	3.17	-0.95	6.96	0.006 *	0.009 *
IL-4	1.33	4.55	-2.67	11.90	0.008 *	0.014
IL-6	0.86	3.43	2.75	14.94	0.022	0.027
IL-8/CXCL8	1.87	3.94	0.20	8.25	0.07	0.072
IL-10	6.79	14.29	-4.43	27.19	0.019	0.024
IL-16	32.62	82.96	-19.07	149.55	0.013	0.021
IP-10/CXCL10	-3.82	52.93	16.13	118.54	0.958	0.893
I-TAC/CXCL11	2.22	7.06	3.73	13.73	0.989	0.955
MCP-1/CCL2	12.78	26.27	-5.34	35.48	0.039	0.055
MCP-2/CCL8	9.01	16.58	-2.92	38.04	0.048	0.071
MCP-3/CCL7	13.07	31.37	-7.03	55.42	0.025	0.029
MCP-4/CCL13	-3.31	25.83	3.02	37.95	0.377	0.418
MDC/CCL22	93.18	244.23	-47.75	453.81	0.004 *	0.005 *
MIF	-1711.46	5475.63	-829.75	3572.37	0.436	0.464
MIG/CXCL9	52.40	128.26	-3.84	200.65	0.016	0.019
MIP-1α/CCL3	0.72	1.66	-0.52	2.67	0.013	0.013
MIP-1δ/CCL15	-300.03	2502.48	515.34	4045.71	0.505	0.368
MIP-3α/CCL20	-16.84	61.17	-4.44	45.65	0.832	0.991
MIP-3β/CCL19	97.36	212.33	-77.06	343.62	0.018	0.024
MPIF-1/CCL23	-16.10	95.08	-29.21	177.39	0.26	0.214
SCYB16/CXCL16	-11.35	114.84	30.26	203.08	0.083	0.11
SDF-1α+β/CXCL12	103.67	198.81	-49.25	409.72	0 *	0.001 *
TARC/CCL17	29.45	72.26	-20.89	96.02	0.022	0.03

TECK/CCL25	54.31	180.75	-57.17	354.85	0.019	0.026
TNFα	4.91	8.98	0.09	18.93	0.03	0.036

Significance taken as * p < 0.01.

Supplementary table II. Metabolic, haemodynamic and other characteristics of patients

	**Baseline**				
	**Placebo (n=13)**		**Metformin (n=20)**		**p value**
Sex	Male 12; Female 1		Male 16; Female 4		
	**Average**	**SD**	**Average**	**SD**	
Age	64.23	6.99	62.70	7.04	0.408
**Metabolism Parameters**					
Body Mass Index	29.37	5.11	30.19	5.23	0.319
Insulin (mU/L)	22.63	11.54	27.47	15.29	0.144
Glucose (mmol/L)	5.37	0.42	5.61	0.66	0.331
Fasting Insulin Resistance Index (log)	4.90	2.57	6.32	3.92	0.134
**Severity of Heart Failure**					
Brain Natriuretic Peptide (pg/ml)	116.27	131.98	139.09	197.95	0.750
Ejection Fraction (%)	28.82	8.29	37.29	7.93	0.016
**Haemodynamic Conditions**					
Resting Systolic Blood Pressure (mmHg)	116.54	21.05	107.60	9.68	0.101
Resting Diastolic Blood Pressure (mmHg)	75.15	10.38	69.35	7.15	0.178
Resting Heart Rate	70.08	21.22	68.60	14.44	0.992
Peak VO_2 (mL/kg/min)	18.02	5.94	19.72	4.75	0.776
VE/VCO_2 slope	30.65	5.31	31.64	6.03	0.244
Total exercise duration (s)	954.15	355.64	1063.80	204.51	0.469

	Change after 4 months metformin treatment				
	Placebo (n=13)		Metformin (n=20)		p value
	Average	SD	Average	SD	
**Metabolism Parameters**					
Body Mass Index	0.46	0.76	-1.16	1.08	0.000
Insulin (mU/L)	0.92	7.39	-6.39	9.19	0.044
Glucose (mmol/L)	-0.02	0.75	-0.29	0.50	0.042
Fasting Insulin Resistance Index (log)	0.26	1.77	-1.81	2.53	0.029
**Severity of Heart Failure**					
Brain Natriuretic Peptide (pg/ml)	17.26	122.33	-22.24	91.36	0.383
Ejection Fraction (%)	-0.89	2.52	-1.03	3.61	0.681
**Haemodynamic Conditions**					
Resting Systolic Blood Pressure (mmHg)	-7.15	16.18	0.80	14.63	0.049
Resting Diastolic Blood Pressure (mmHg)	-4.69	9.22	-0.45	7.84	0.177
Resting Heart Rate	1.00	11.73	2.70	9.95	0.451
Peak VO_2 (mL/kg/min)	1.25	4.59	-0.91	2.73	0.334
VE/VCO_2 slope	1.86	10.57	-4.52	5.37	0.029
Total exercise duration (s)	7.08	92.03	-22.75	99.45	0.671

Supplementary figure legends

Supplementary Figure I. Densitometry of blots in main figure 1

Densitometry was carried out as described in the methods to quantify data obtained in western blots. Bars significantly different from the respective control treatment (+/- TNFα) are shown, ***p<0.001, **p<0.01, *p<0.05. N=3 except for IκBa and pACC, N=4

Supplementary Figure II. Densitometry of blots in main figure 2

Densitometry was carried out as described in the methods to quantify data obtained in western blots. In experiments comparing wild-type (WT) and knockout (KO) genotype, black bars denote WT genotype, grey bars denote KO genotype. Bars significantly different from the respective control treatment (+/- TNFα) are shown,

***p<0.001, **p<0.01, *p<0.05, except in knockout experiments, where significance between genotypes is depicted. N=3

Supplementary Figure III. Effect of metformin on gene expression in macrophages: genes regulated in hepatocytes
Macrophages were treated with or without 100ng/ml LPS +/- 2mM metformin for 8h followed by cell lysis, RNA extraction and preparation of cDNA for RTPCR using primer sets for individual genes shown, as described in the methods. Bars significantly different from control treatment, or between two annotated bars are shown, ***p<0.001, *p<0.05

Supplementary Figure IV. Effect of metformin on gene expression in macrophages and hepatocytes: genes changed by metformin in human plasma
Macrophages *(a,b)* were treated with/without 100ng/ml LPS +/- 2mM metformin, while hepatocytes *(c)* were treated with/without 10ng/ml TNFα +/- 2mM metformin or 10mM BI605906 as shown, for 8h followed by cell lysis, RNA extraction and preparation of cDNA for RTPCR using primer sets for individual genes shown, as described in the methods. Bars significantly different from control treatment, or between two annotated bars are shown, ***p<0.001, **p<0.01, *p<0.05

Supplementary Figure V. Densitometry of blots in main figure 5
Densitometry was carried out as described in the methods to quantify data obtained in western blots. In experiments comparing wild-type (WT) and knockout (KO) genotype, black bars denote WT genotype, grey bars denote KO genotype. Bars significantly different from the respective control treatment (+/- TNFα) are shown, ***p<0.001, **p<0.01, *p<0.05, except in knockout experiments, where significance between genotypes is depicted. N=3

Supplementary Fig. I

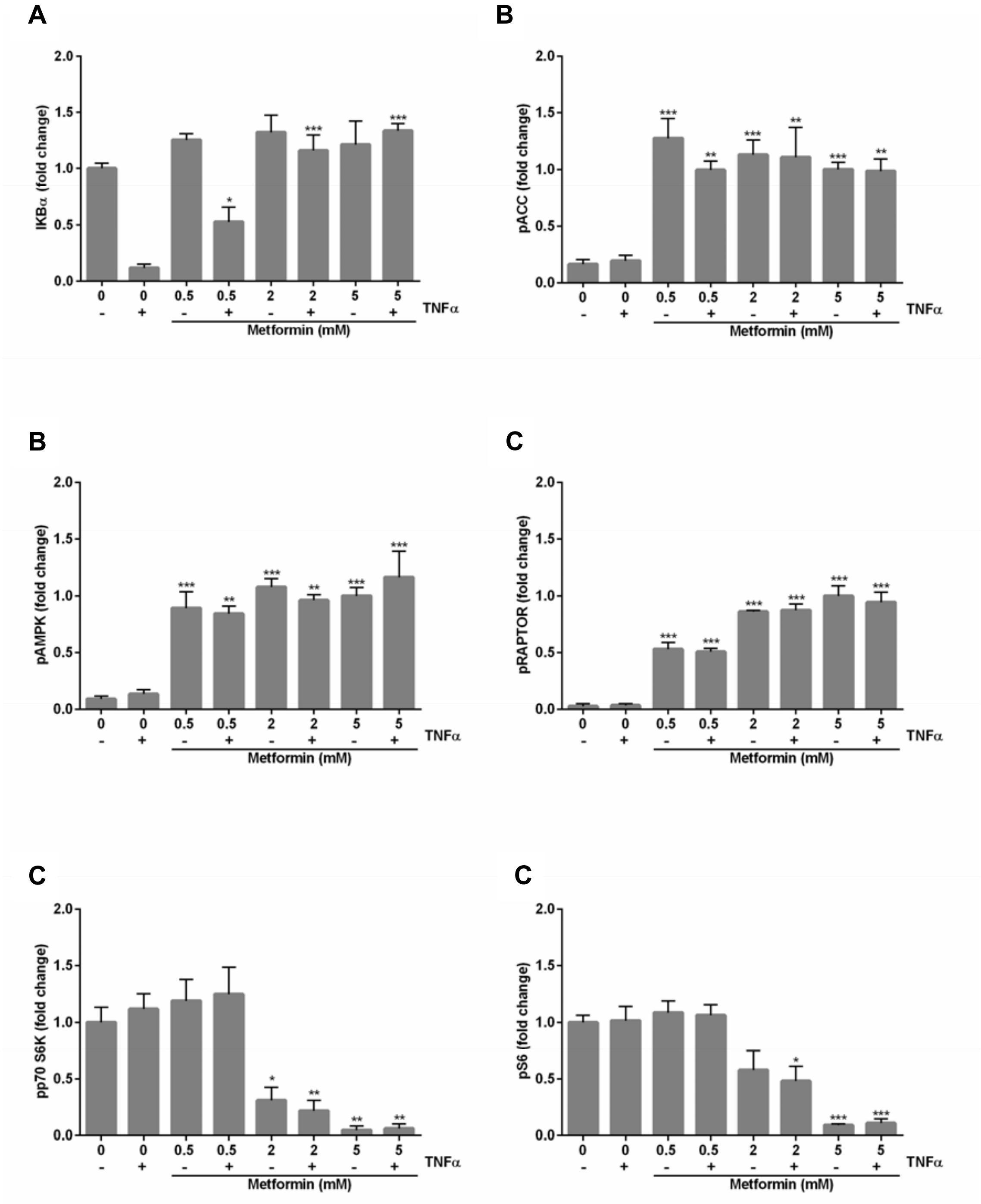

Supplementary Fig. I

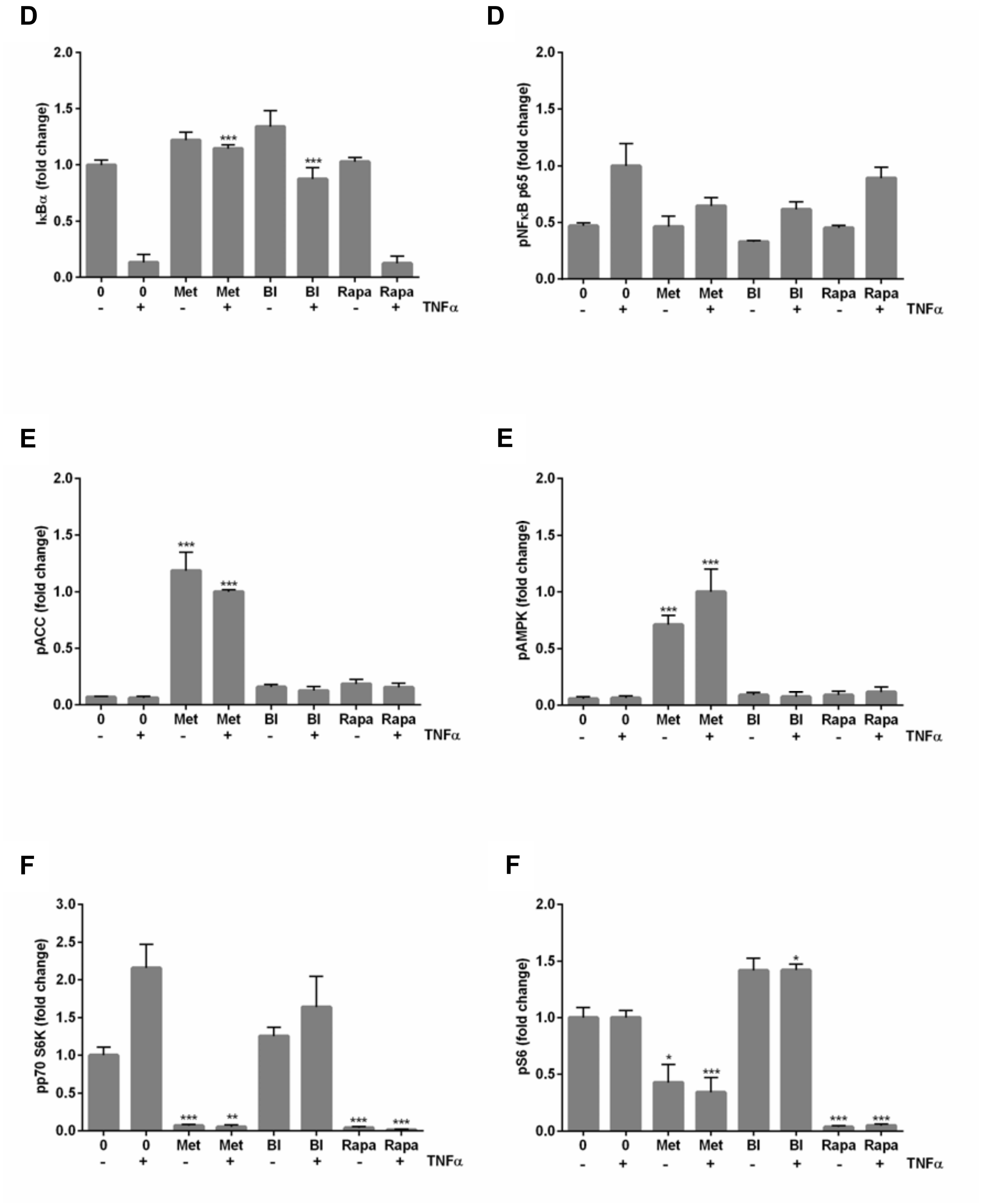

Supplementary Fig. II

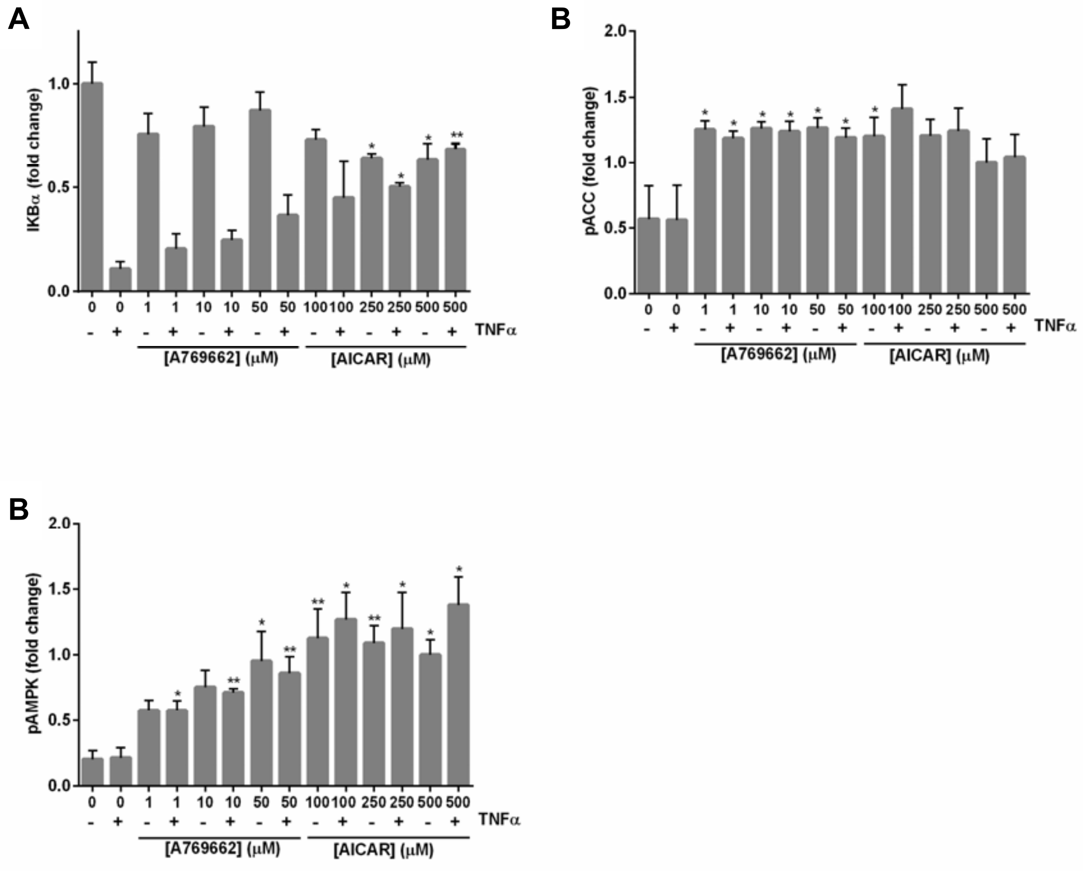

Supplementary Fig. II

C

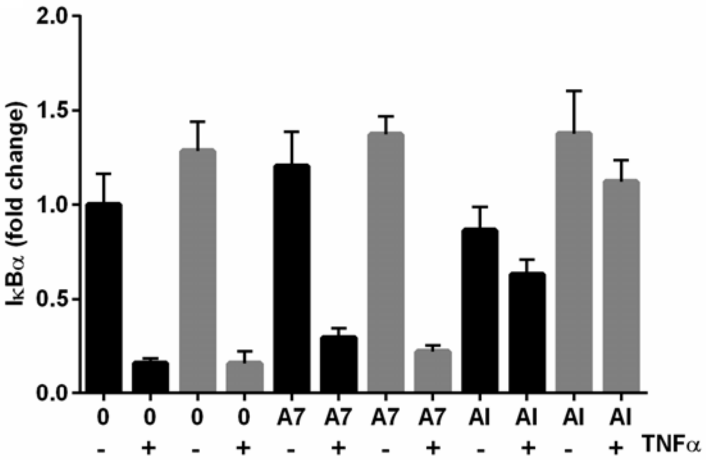

C

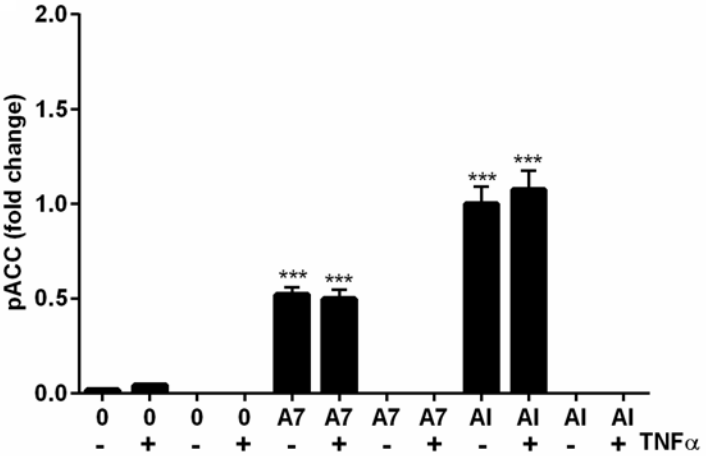

C

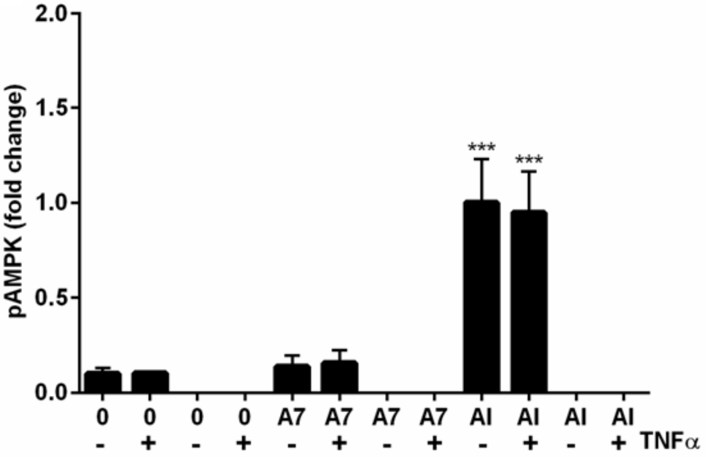

Supplementary Fig. II

D

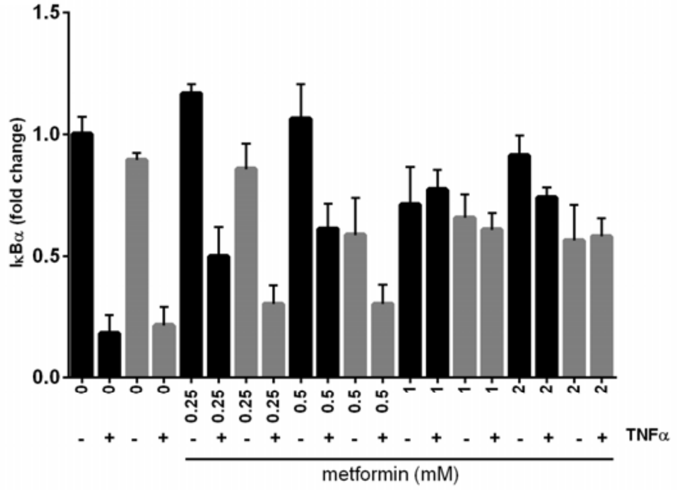

D

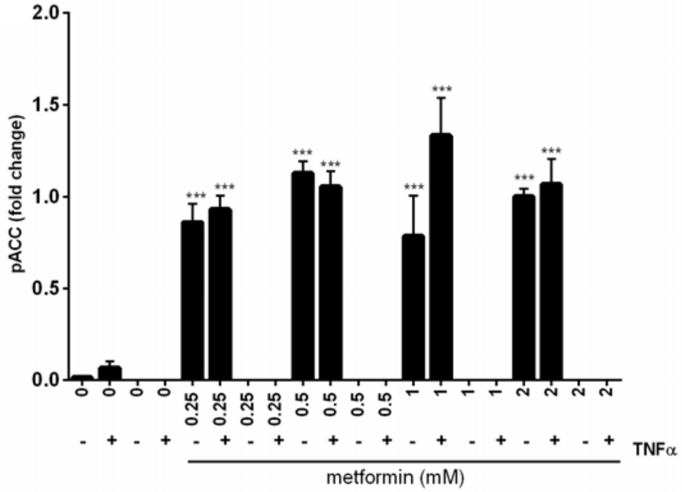

D

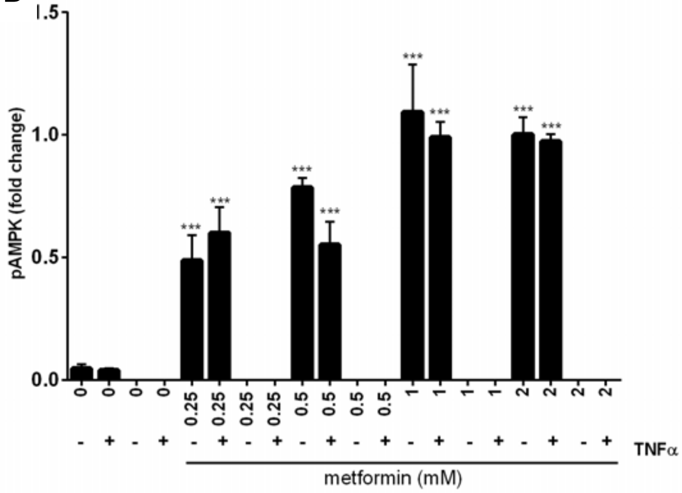

Supplementary Fig. III

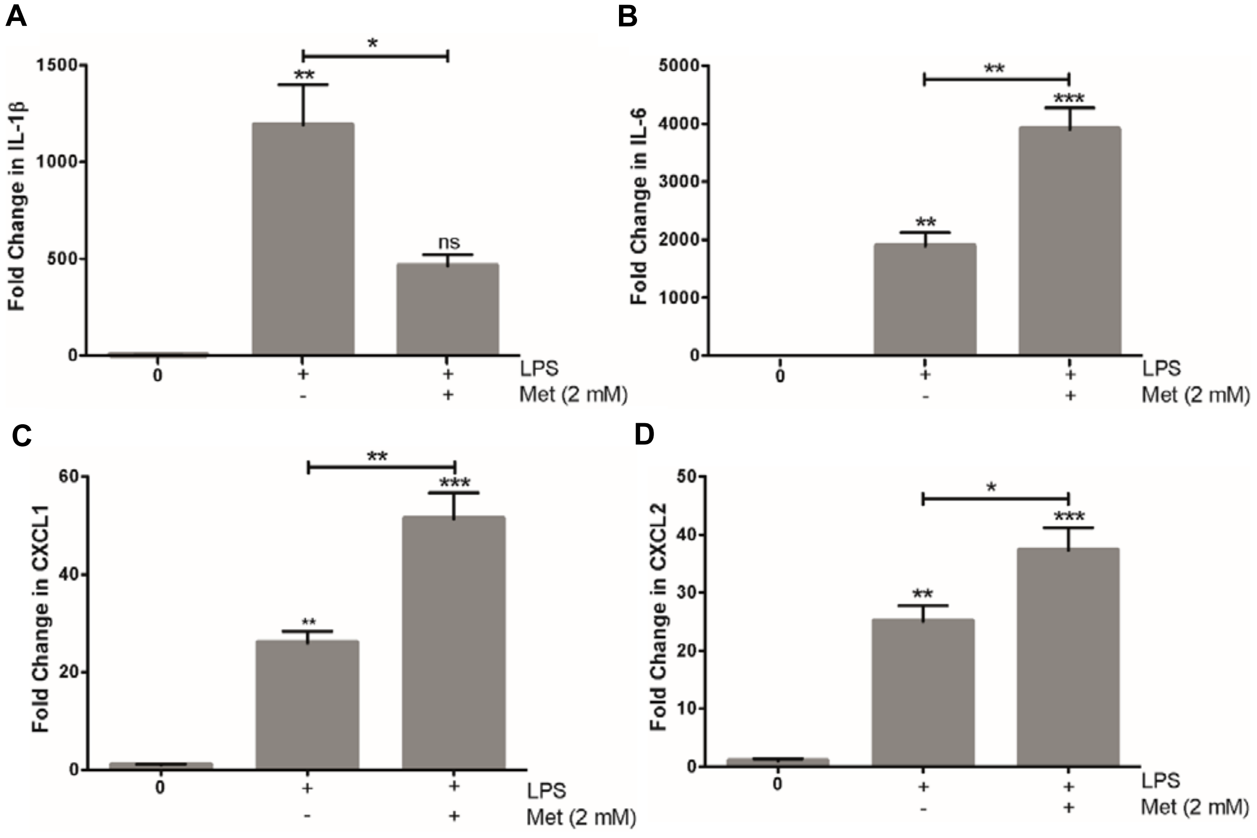

Supplementary Fig. IV

MACROPHAGES

A **B**

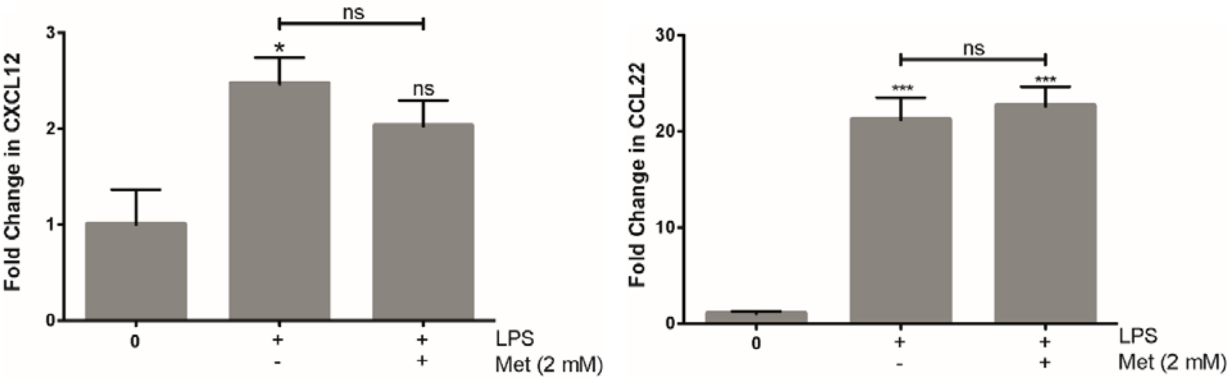

HEPATOCYTES

C

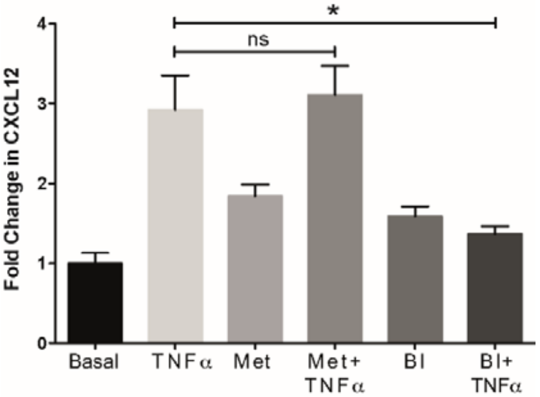

Supplementary Fig. V

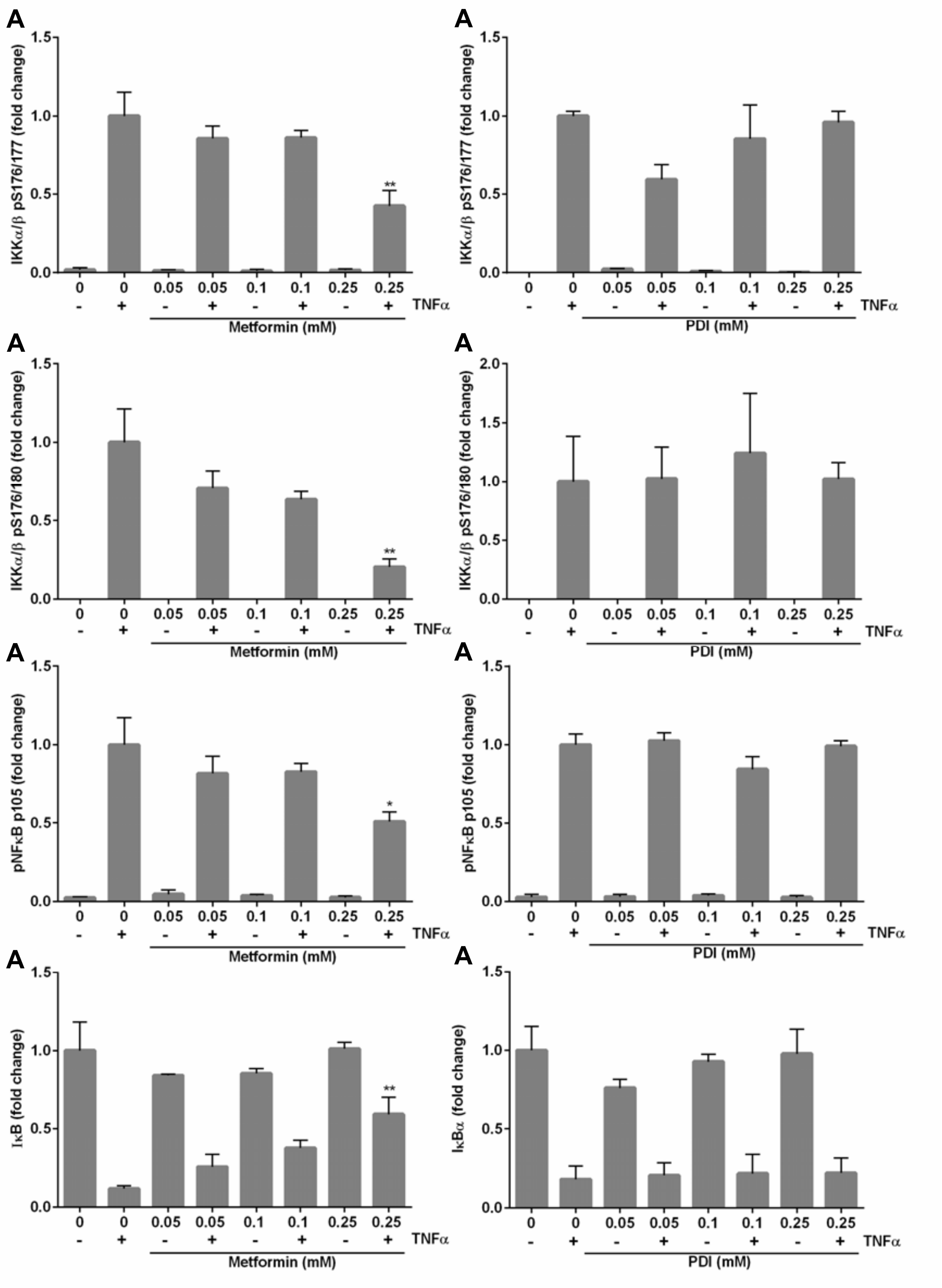

Supplementary Fig. V

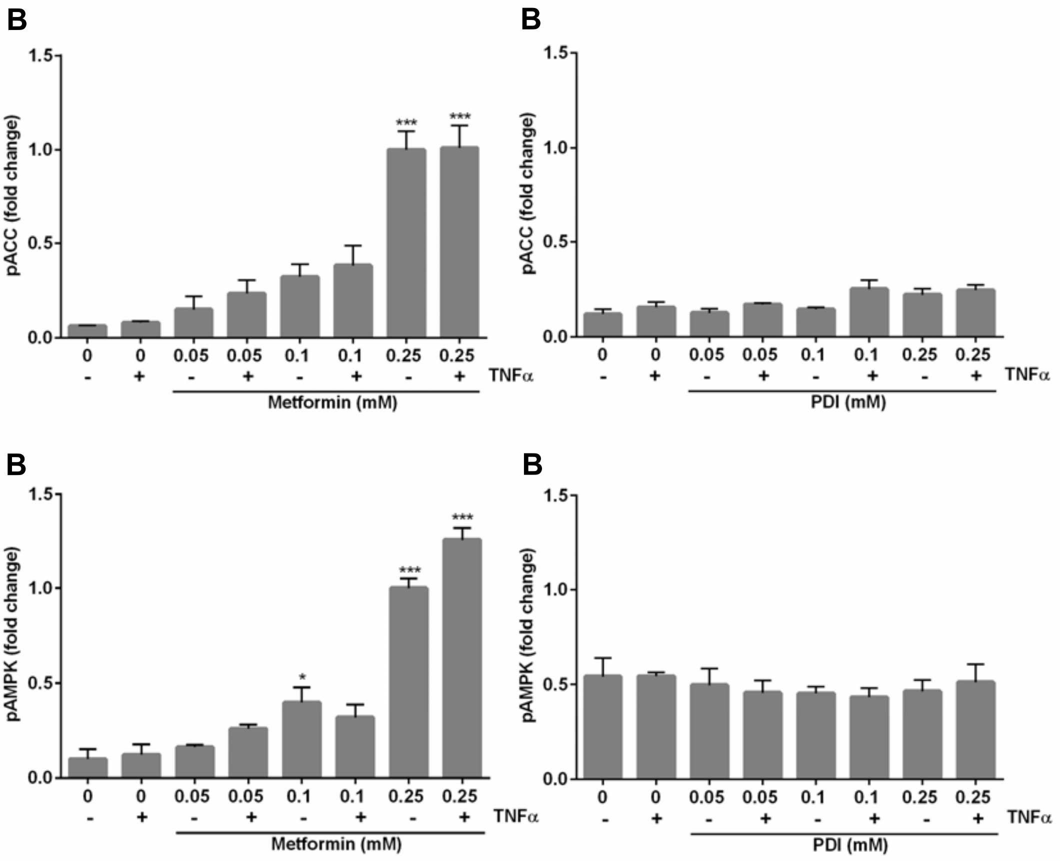

Supplementary Fig. V

C

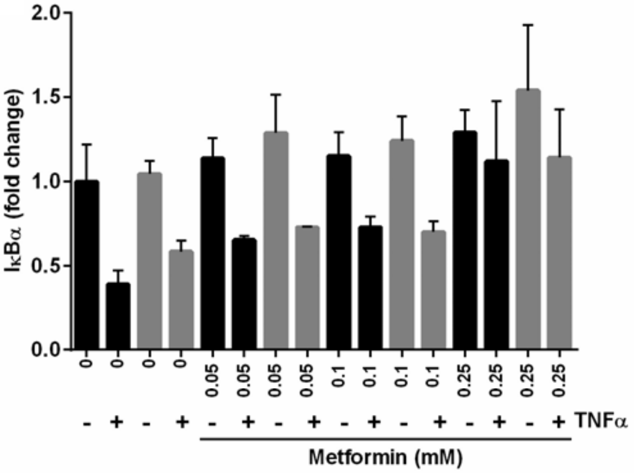

C

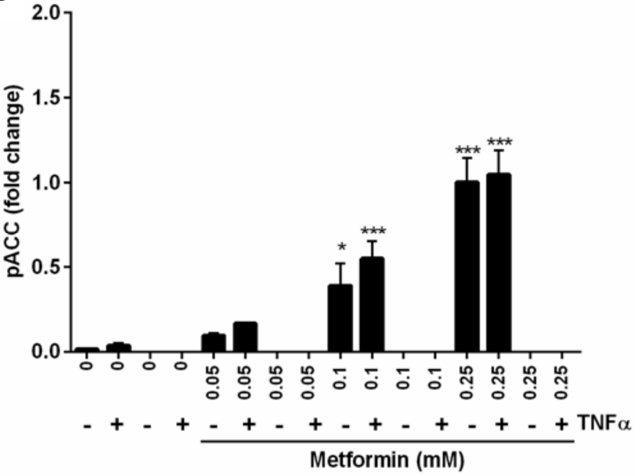

C

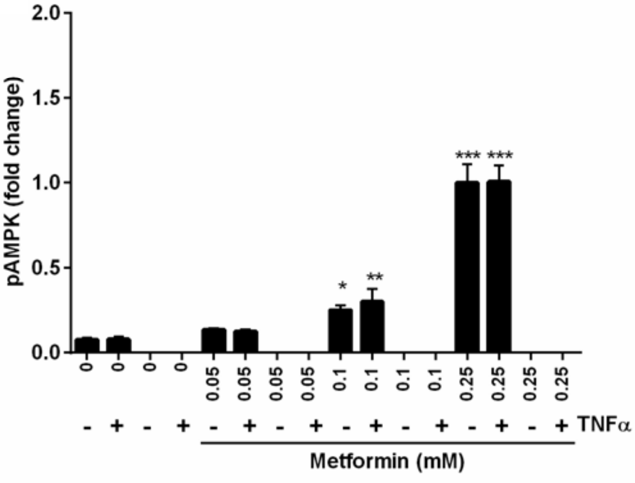

Supplementary Fig. V

D

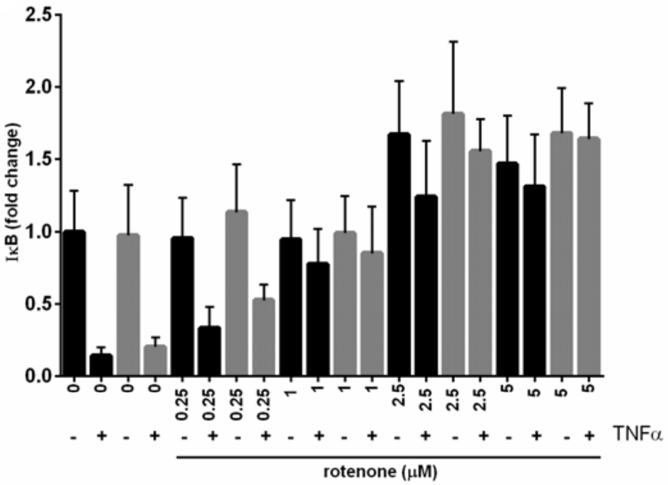

D

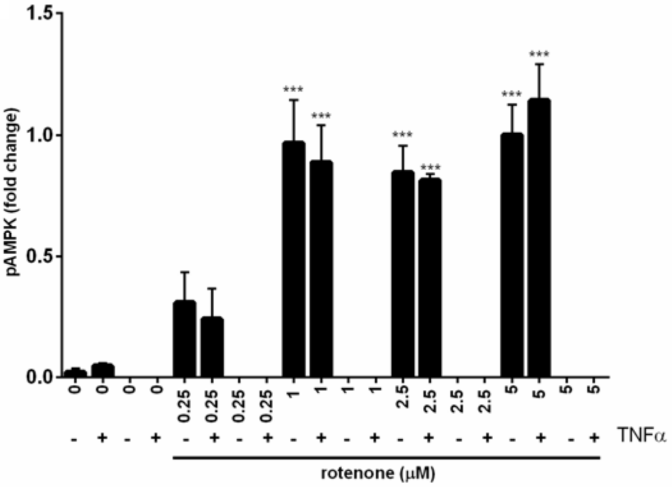

D

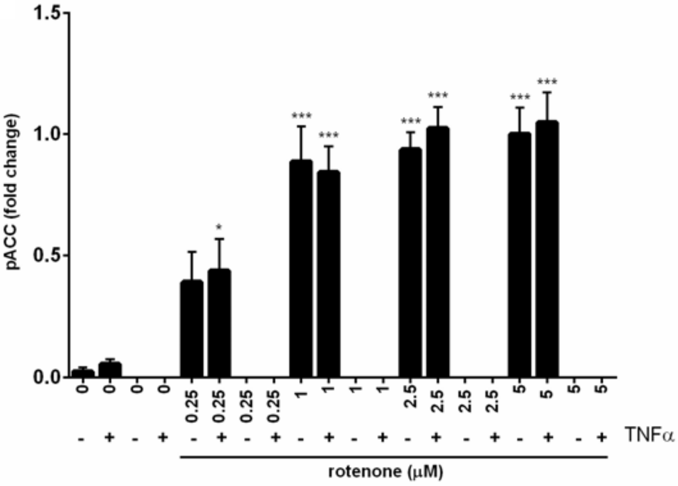

Supplement: Supplementary file 2 [file res-119-652-s002.pdf]
